# Root traits are strongly associated with nitrogen uptake efficiency in contrasting rapeseed genotypes

**DOI:** 10.1186/s12870-026-09682-5

**Published:** 2026-08-05

**Authors:** Xiao Guo, Tingting Dai, Ji Luo, Qian Luo, Yanli Chen

**Affiliations:** 1 Research Center of Agricultural Economy, Sichuan University of Science & Engineering, Zigong, Sichuan China; 2 Sichuan Provincial Engineering Research Center of Resource Utilization for Agricultural and Forestry Wastes, Chengdu Normal University, Chengdu, Sichuan China; 3 Shipping Logistics Collaborative Innovation Center in the Upper reaches of Yangtze River, Yibin University, Yibin, Sichuan China

**Keywords:** Rapeseed, Nitrogen uptake efficiency, N-efficient-genotype, Root indexes

## Abstract

Screening the higher nitrogen (N) uptake efficiency (NUpE) rapeseed (*Brassica napus* L.) genotype (N-efficient-genotype) and exploring the N efficient uptake mechanism could be an ideal way to meet the environmentally use of valuable N and the increasing demand for edible oils. In this study, we selected a N-efficient-genotype and a N-inefficient-genotype to dissect the differences in root physiological indexes under a pot experiment and a hydroponic culture. Results showed that during the high and low N levels, root biomass was the main factor affecting NUpE at the pot experiment. Compared to the N-inefficient-genotype, N-efficient-genotype displayed respectively 39.72%, 31.52%, 39.94%, 41.28% and 54.80% higher root biomass, N content, root length, root surface area and Vmax, along with 36.86%,48.68%, 134.14%, 72.71%, nitrate reductase (NR), glutamine synthetase (GS), catalase (CAT) and glutathione (GSH) activity. These were accompanied by a significant increase (*P* < 0.05) in *BnNRT1.1* (7.04 fold), *BnNRT1.5* (4.66 fold), *BnNRT2.1* (8.63 fold) and *BnNRT2.5* (12.13 fold), which may result in the greater N uptake ability than did the N-inefficient-genotype under the hydroponic culture. Our results indicate that higher root indexes for the N-efficient-genotype could uptake more N to support the photosynthesis sustained and seed development, and would achieve the larger NUpE and NUE in rapeseed genotypes.

**Supplementary Information:**

The online version contains supplementary material available at https://doi.org/10.1186/s12870-026-09682-5.

## Introduction

 As the major advance to increase productivity of land [1], invention of Haber-Bosch nitrogen (N) fixation, which generated by fixing atmospheric N_2_ into plant available inorganic N forms, has contributed to high agricultural production to meet the demand from a predicted world population near 10 billion by 2050 [2]. To achieve optimal growth and yield, fields are subjected to massive applications of N fertilizers, but only 25–50% of the applied N could be efficiently uptake by plants [3]. The remaining N in soil would lead to a huge waste of resource with higher production costs and a greater risk from environmental hazards, and such phenomenon have raised people’s concerns. In 2015 and 2022, “Action Plan for Nitrogen Fertilizer Reduction” by the Ministry of Agriculture and Rural Affairs of the People’s Republic China was announced to improve N use efficiency (NUE) [4]. It was reported that NUE has comprised two key major components: N utilization efficiency (NUtE) (grain yield / plant N uptake) and N uptake efficiency (NUpE) (plant N uptake / soil available N) [5]. Theoretically, increasing NUE can be achieved by increasing either NUtE or NUpE, or one or more of the two components together [4].

Rapeseed (*Brassica napus* L.), which is the key oilseed crops, provides more than 15% of the essential edible oil for human consumption [6]. During the last several decades, N fertilizer application has remarkably enhanced both the quantity and quality of rapeseed [7–9]. Compared with the other four largest crops (rice, maize, wheat and soybean), N fertilizer requirement of rapeseed (150–250 kg ha^− 1^) is second only to that of soybeans, but has been often described as a low-NUE crop species [10]. Additionally, literature showed that NUE of rapeseed is from 15 to 20 kg seeds kg^− 1^ N, which is approximately half that of cereals [11]. Therefore, enhancing NUE without yield losses has become a critical objective in sustainable agriculture for crop physiologists and plant breeders. For most plants, efficient uptake of N from soil is the critical step toward successful N acquisition and remobilization, and ultimately boost NUE [12]. Preliminary study in our group [13] found that the leaf (high N level) and stem (low N level) remobilized the most N for seed development, and strengthened the mechanism to enhance nitrate uptake and assimilation for seed yield and NUE stability [14]. In addition, available literatures suggested that N uptake of plant is related to root traits, such as root characteristics, root N metabolism enzyme activity, root senescence-related enzyme activity and root nitrate transporter activity [15, 16].

Root characteristics is not only an important organ for plants to absorb water and nutrients but also the first organ to sense changes in rhizosphere environmental conditions [17]. Li et al. [17] reported that N-efficient plant in the vegetative growth stage had higher root tips, root surface area and root volume, resulting in the greater N uptake and utilization efficiencies than the N-inefficient rapeseed genotype. Nitrate is one of the major forms of N uptake, and nitrate uptake by plants mainly mediated by nitrate transporters 1 (NRT1) and nitrate transporters 2 (NRT2) according to the external nitrate concentration (NRT1 for higher than 1 mmol L^− 1^ and NRT2 for lower than 1 mmol L^− 1^) [18, 19]. GL Wang, GD Ding, FS Xu, HM Cai, J Zou and XS Ye [20] found that the expression levels of *BnNRT1.1*, *BnNRT2.5*, *BnNRT2.6* and *BnNRT2.7* in roots of N-efficient rapeseed seedlings were significantly higher than those of N-inefficient plants. In summary, we believed there would be a relationship between root traits and NUE in rapeseed [16] and other crops [14, 21], and these studies could support the plausibility of root traits as a key driver of NUpE and NUE, while also highlighting the complexity of the trait. Therefor, screening the high and low NUpE rapeseed genotypes, and verification the biological root mechanism between them could be an ideal way to improve NUE.

According to the definition [22], NUpE comprises 10 plant indicators: seed biomass, silique husk biomass, stem biomass, leaf biomass, root biomass, seed N content, silique husk N content, stem N content, leaf N content and root N content. Previous researches focused on the indicators related to NUpE at seedling stage [23, 24], but literatures believed that the growth attribute of rapeseed plants were different between the seedling and maturity stage [2, 24]. Therefore, exploring the key plant indicators affecting NUpE in pod-filling stage (BBCH 75, 50% of pods have reached final size) [25] could be more accurate. On the other hand, the existing studies have reported the relationship between root morphological parameters and NUE in detail under field or pot experiments [26, 27], which could not avoid data deviations caused by root loss. Investigating the relationship between root indexes and NUpE under hydroponic culture might be a good choice.

Based on this, we have firstly used 50 rapeseed genotypes and conducted a pot experiment to analyse the relationship between plant indicators and NUpE. And then, we hypothesised the root biomass was the factor that has the greatest influence on NUpE. Finally, a high (N-efficient-genotype) and a low NUpE (N-inefficient-genotype) rapeseed genotypes were selected to explore the differences in root physiological indexes in detail under hydroponic culture at pod-filling stage. The novelty of this study lay in (i) the systematic integration of pot-based population screening with hydroponic physiological dissection to identify root biomass as the primary determinant of NUpE at the pod-filling stage and (ii) the comprehensive characterization of root morphological, kinetic, enzymatic and relative RNA expression in contrasting NUpE genotypes under controlled conditions. This multi-level approach could provide a way for root-targeted breeding strategies to improve NUE in rapeseed.

## Materials and methods

### Pot experiment

#### Plant materials

The 50 rapeseed genotypes were obtained from the Oil Crops Research Institute of the Chinese Academy of Agricultural Sciences, and their origin, ecotype and breeding period have been described in HY He, R Yang, YJ Li, AS Ma, LQ Cao, XM Wu, BY Chen, H Tian and YJ Gao [28].

#### Growing conditions

The experimental design of pot experiment was conducted at Yangling district, Shaanxi province, China (34° 240 N, 108° 080 E) from 2017 to 2018, and each pot contained 9 kg of air-dried red loess soil (0–20 cm) with pH value of 7.51, 7.26 g kg^− 1^ organic matter, 0.73 g kg^− 1^ of total N, 20.95 mg of available N kg^− 1^, 8.25 mg of Olsen-P kg^− 1^ and 76.92 mg of available K kg^− 1^. There were two N levels, high (0.30 g of N kg^− 1^ dry soil) and low N level (0.10 g of N kg^− 1^ dry soil) with 4 biological replicates (*n* = 4). Seeds of the 50 rapeseed genotypes were sown on 15 Oct. 2017, and only one single plant was retained in each pot on 20 Jan. 2018 until maturity on 1 May 2018. Throughout the experiment, all of the pots were rotated once a week to avoid positional effect.

#### Sampling and measurements

At maturity (BBCH 89), each rapeseed plant was harvested and divided into roots, stem, leaves, silique husks and seeds to place in an oven at 70 °C for 24 h to constant weight. Afterward, the biomass of the rapeseed plant organs was calculated. And then, the plant organs were ground to a homogeneous powder (via 100-mesh beads) prior to analysis the N content using the Kjeldahl method. Finally, NUpE was estimated with the following equation: NUpE (g g^− 1^) = shoot N accumulation / N supply [22] (Table S1 and 2).

### Hydroponic culture

#### Plant materials

Two rapeseed genotypes were selected as the experimental materials based on their contrasting NUpE, including one N-efficient-genotype (No. 44) and one N-inefficient-genotype (No. 41) (Table S1 and 2).

#### Growing conditions

The experimental design of hydroponic culture was conducted at Sichuan University of Science & Engineering, Sichuan province, China (29° 335 N, 104° 766 E) from 2024 to 2025. Seeds of the N-efficient-genotype and N-inefficient-genotype with uniform size were soaked with 1% H_2_O_2_ solution for 10 min and rinsed 3 times with distilled water, and then to put them on a moistened filter paper for germinating. After 1 week, 48 the healthy and uniform rapeseed seedlings (24 for N-efficient-genotype and 24 for N-inefficient-genotype) were transplanted carefully into 12 black plastic boxes (40 cm length × 30 cm width × 20 cm height; 6 per N level × 2 genotypes), and each box contained 4 plants.

All of the black plastic boxes were divided into two groups: high N (10 mM KNO_3_) and low N (1 mM KNO_3_) treatments, with 6 boxes in each of the high and low N levels. After that, all of the rapeseed seedlings were cultured in distilled water for the first week, 1 / 4 strength of modified Hoagland solution (Table S3) in the second week, 1 / 2 strength for the third week, and received full strength of nutrient solution till harvesting (BBCH 75). This experiment followed a randomized complete block design with 3 biological replicates (*n* = 3) and the nutrient solution was replenished 3 days with pH 5.8, and all of the 12 black plastic boxes in greenhouse were rotated once a week to avoid positional effect. All the rapeseed seedlings were grown under the following conditions: day / night mean temperature of 25 / 16 °C, relative humidity of 65%, and light intensity of 400 µmol m^− 2^ s^− 1^, with a 12 h photoperiod.

#### Sampling and measurements

At pod-filling stage (BBCH 75), all of the 48 rapeseed plants were divided into 2 groups. Group 1 (24 plants; 12 for N-efficient-genotype and 12 for N-inefficient-genotype) was to measure the root biomass and N content, root characteristics and nitrate uptake kinetic parameters. Group 2 (24 plants; 12 for N-efficient-genotype and 12 for N-inefficient-genotype) was to measure N metabolism enzyme activity, root senescence-related enzyme activity and root relative RNA expression. Treating each box as 1 biological replicate ensured that the error term reflected true among‑box variation, not within‑box dependency. Since all plants in the same box share the same nutrient solution, they were not independent.

Group 1: (1) the roots of the first 3 N-efficient-genotype and 3 N-inefficient-genotype plants were cut to measure the biomass and N content; (2) the root length, root surface area, root volume, root vigor and root diameter of the second 3 N-efficient-genotype and 3 N-inefficient-genotype plants were cut to measure using the root image analysis scanner and WinRHIZO Pro software (Regent Instruments Inc., Quebec, QC, Canada); (3) the roots of the third 3 N-efficient-genotype and 3 N-inefficient-genotype plants were preincubated for 24 h in a 500 mL beaker with N-free growth medium for NO_3_^−^ uptake, and then the Vmax and Km were measured [15]; (4) the remaining 3 N-efficient-genotype and 3 N-inefficient-genotype plants were used as the supplemental materials for the above 3 measurement processes.

Group 2: The roots of the 24 rapeseed plants were cut and immediately ground in liquid N. (1) the activities of the root N metabolism enzyme (nitrate reductase, NR; glutamine synthetase, GS; glutamate synthetase, GOGAT; glutamate dehydrogenase, GDH) between the first 3 N-efficient-genotype and 3 N-inefficient-genotype plants were measured according to the method of X Guo, XL Li, J Luo and PY Wang [2]; (2) the roots of the second 3 N-efficient-genotype and 3 N-inefficient-genotype plants were used to measure root senescence-related enzyme (catalase, CAT; peroxidase, POD; glutathione, GSH; ascorbate peroxidase, APX; superoxide dismutase, SOD) activity and soluble sugar [29, 30]; (3) the total RNA was extracted using an EZNATM Plant RNA Kit (Omega Bio-Tek Inc., Norcross, GA, USA), and used for cDNA synthesis by TransScript^®^ First-Strand cDNA Synthesis SuperMix (TransGen Biotech, Beijing, China) according to the manufacturer’s instructions. The relative expression of *Actin*, *NRT1.1*,* NRT1.5*,* NRT1.8*,* NRT2.1*,* NRT2.5* and *NRT2.6* genes (Table S4) were determined by quantitative RT-PCR using the SYBR Green Real-Time PCR Master Mix Kit (TOYOBO, Japan). For qRT‑PCR, each biological replicate was measured in 3 technical replicates, and the mean of the technical replicates was used for downstream calculations. The relative quantities of the transcripts were calculated using the standard curve method and N-inefficient-genotype was used as reference; (4) the remaining 3 N-efficient-genotype and 3 N-inefficient-genotype plants were as described above in Group 1.

### Statistical analysis

The pot experiment employed 4 biological replicates per genotype × N level (1 plant per pot), and all the pots were rotated weekly to minimize positional effects. For the hydroponic culture, each black plastic box (40 × 30 × 20 cm) containing four plants was considered one biological replicate (*n* = 3 per genotype × N level), and all measurements from the 4 plants in the same box were averaged. All the data were subjected to analysis of variance. Estimating of correlation coefficients and testing of significance were performed using the SPSS version 17.0. When the analysis showed significance, mean comparisons were made according to the least-significant difference test at *P* *≤* 0.05 (LSD_0.05_). Principal component analysis was performed to estimate the contribution of the measured plant traits to NUpE by using Canoco 5.0 software. All the figures were created using Origin 9.0 software.

## Results

### Pot experiment identified root biomass as the core determinant of NUpE

To quantify the relative importance of each trait in explaining NUpE variation, we performed single-variable linear regressions for each of the 11 measured indicators under both high and N levels (Table 1). Under high N, root biomass explained the largest proportion of NUpE variance (*R*² = 0.632), followed by seed biomass (*R*² = 0.581) and stem biomass (*R*² = 0.547). Under low N, seed biomass ranked first (*R*² = 0.670), closely followed by root biomass (*R*² = 0.653) and stem biomass (*R*² = 0.618).

**Table 1:** The variation in NUpE explained by each trait under both high and low N levels

*N* levels	Traits	*R* ^2^	Rank
High N	Root biomass	0.632	1
	Seed biomass	0.581	2
	Stem biomass	0.547	3
	Total biomass	0.546	4
	Silique husk biomass	0.418	5
	Leaf biomass	0.303	6
	Seed N content	0.185	7
	Root N content	0.025	8
	Stem N content	0.021	9
	Leaf N content	0.014	10
Low N	Root biomass	0.670	1
	Seed biomass	0.653	2
	Stem biomass	0.618	3
	Total biomass	0.605	4
	Silique husk biomass	0.442	5
	Leaf biomass	0.281	6
	Seed N content	0.089	7
	Root N content	0.051	8
	Stem N content	0.038	9
	Leaf N content	0.024	10

**Fig. 1:**
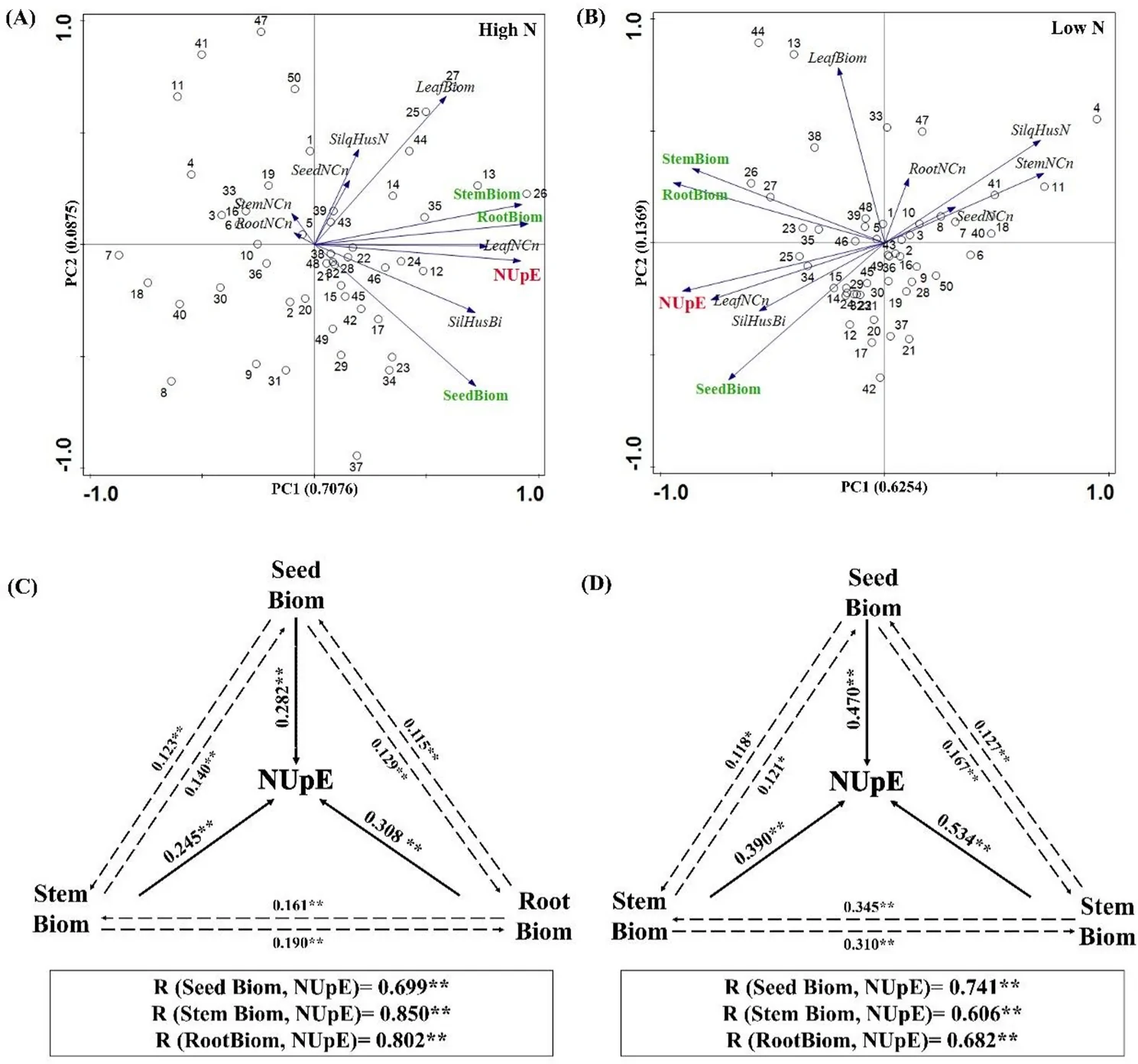
Principal component analysis in high N (**A**) and low N (**B**) and the path coefficient for NUpE, seed biomass, stem biomass and root biomass in high N (**C**) and low N (**D**) of the 50 oilseed rape genotypes under pot experiment. SeedBiom, seed biomass; SilHusBi, silique husk biomass; StemBiom, stem biomass; LeafBiom, leaf biomass; RootBiom, root biomass; F, SeedNCn; G, SilgHusN; StemNCn, stem N content; LeafNCn, leaf N content; RootNCn, root N content

Association between traits represented by the first two principal components is depicted (Fig. 1A-B), and principal components explained about 75% of the total variation. The first principal component was mainly spanned by traits of NUpE, seed biomass, silique husk biomass, stem biomass, leaf biomass, root biomass, and leaf N content on the positive side of the scale, while stem N content and root N content spanned on the negative side. Further research indicated three traits (seed biomass, stem biomass and root biomass) showed obviously positive correlations with NUpE, and we continued to explore the correlation between three traits and NUpE.

Path coefficient analysis illustrated that the direct and indirect effects of the three traits (seed biomass, stem biomass and root biomass) on NUpE and their interrelationships (Fig. 1C-D). The seed biomass, stem biomass and root biomass exhibited a positive direct effect on NUpE, and there were also indirect effects on NUpE among the three traits. Results from the values of the direct effects on NUpE, we found the root biomass was the main factor affecting NUpE, and regression analysis (Fig. S1A-B) further proved the correlation between root biomass and NUpE. Based on this, our subsequent research would mainly focus on exploring the differences in root physiological indexes between the contrasting NUpE rapeseed genotypes.

### Hydroponic culture verified root morphological advantages of N-efficient genotypes

**Fig. 2:**
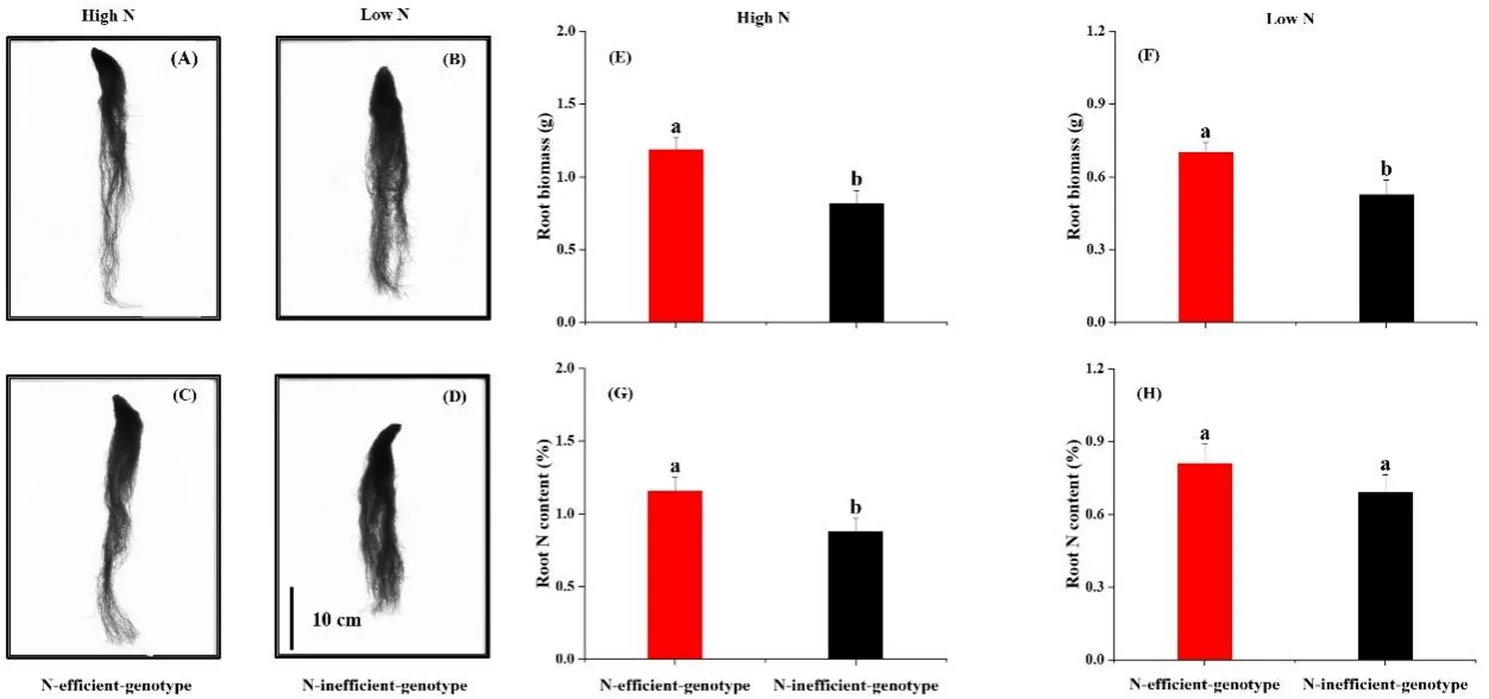
Differences in root phenotype (**A**-**D**), biomass (**E**-**F**) and N content (**G**-**H**) between N-efficient-genotype and N-inefficient-genotype under hydroponic culture. Each bar above the mean value represents standard error of the mean (*n* = 3), and different letters above the bars within a figure indicate significant differences according to the ANOVA-protected LSD_0.05_ test

Under high and low levels, the root biomass of the N-efficient-genotype was obviously higher than that for the N-inefficient-genotype (Fig. 2A-D), respectively 46.25% and 33.19% (Fig. 2E-F). On the other hand, the N-efficient-genotype showed 46.09% and 16.95% higher root N content than did the N-inefficient-genotype (Fig. 2G-H) at the pod-filling stage.

**Fig. 3:**
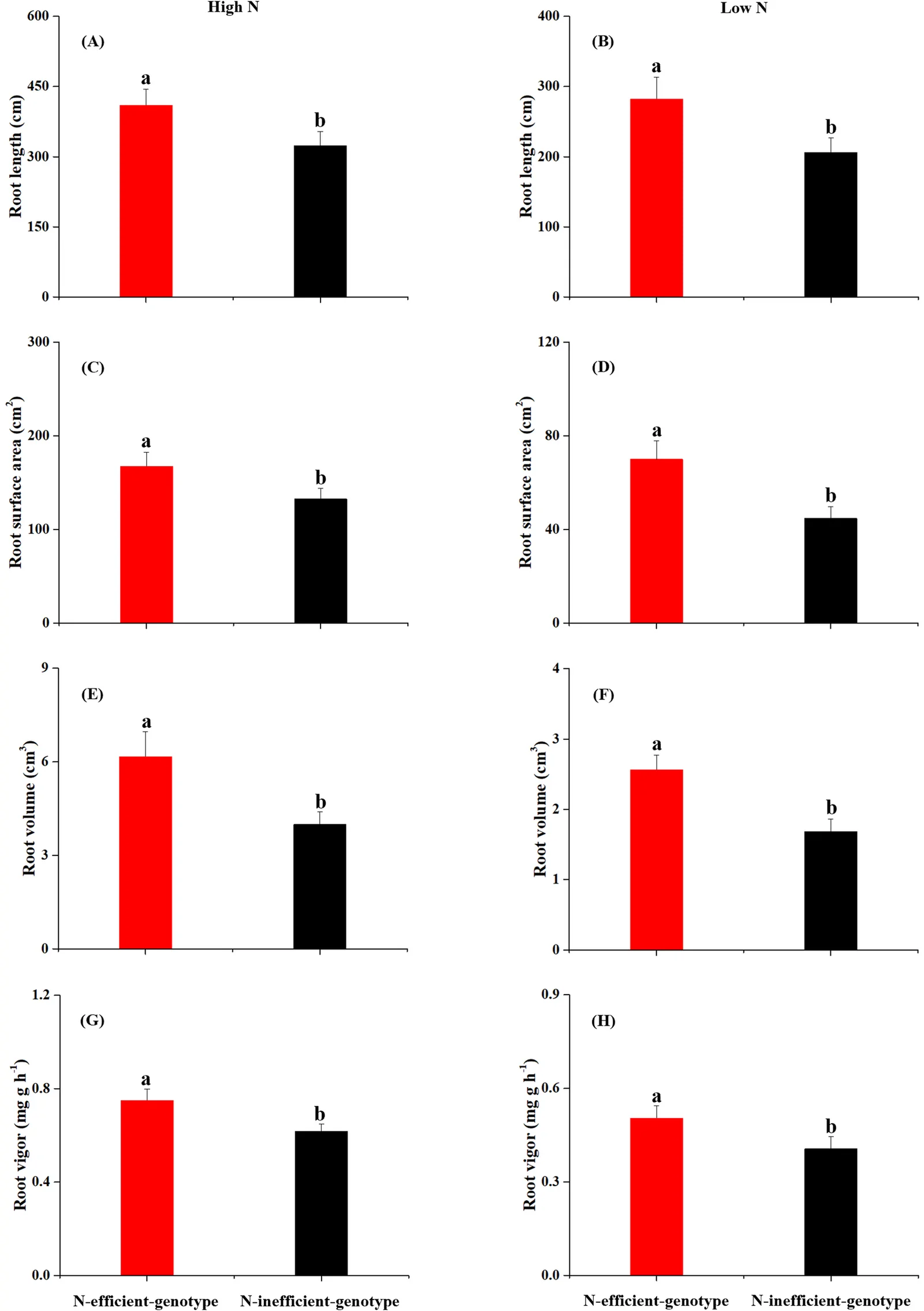
Differences in root characteristics (root length (**A**-**B**); root surface area (**C**-**D**); root volume (**E**-**F**); root vigor (**G**-**H**)) between N-efficient-genotype and N-inefficient-genotype under hydroponic culture. Each bar above the mean value represents standard error of the mean (*n* = 3), and different letters above the bars within a figure indicate significant differences according to the ANOVA-protected LSD_0.05_ test

On average, it was showed that the root length, root surface area, root volume, root vigor and root diameter of the N-efficient-genotype were respectively 39.94% (Fig. 3A-B), 33.94% (Fig. 3C-D), 41.28% (Fig. 3E-F), 10.45% (Fig. 3G-H) and 26.96% (Fig. S2A-B) larger than did the N-inefficient-genotype both at the high and low treatments.

**Fig. 4:**
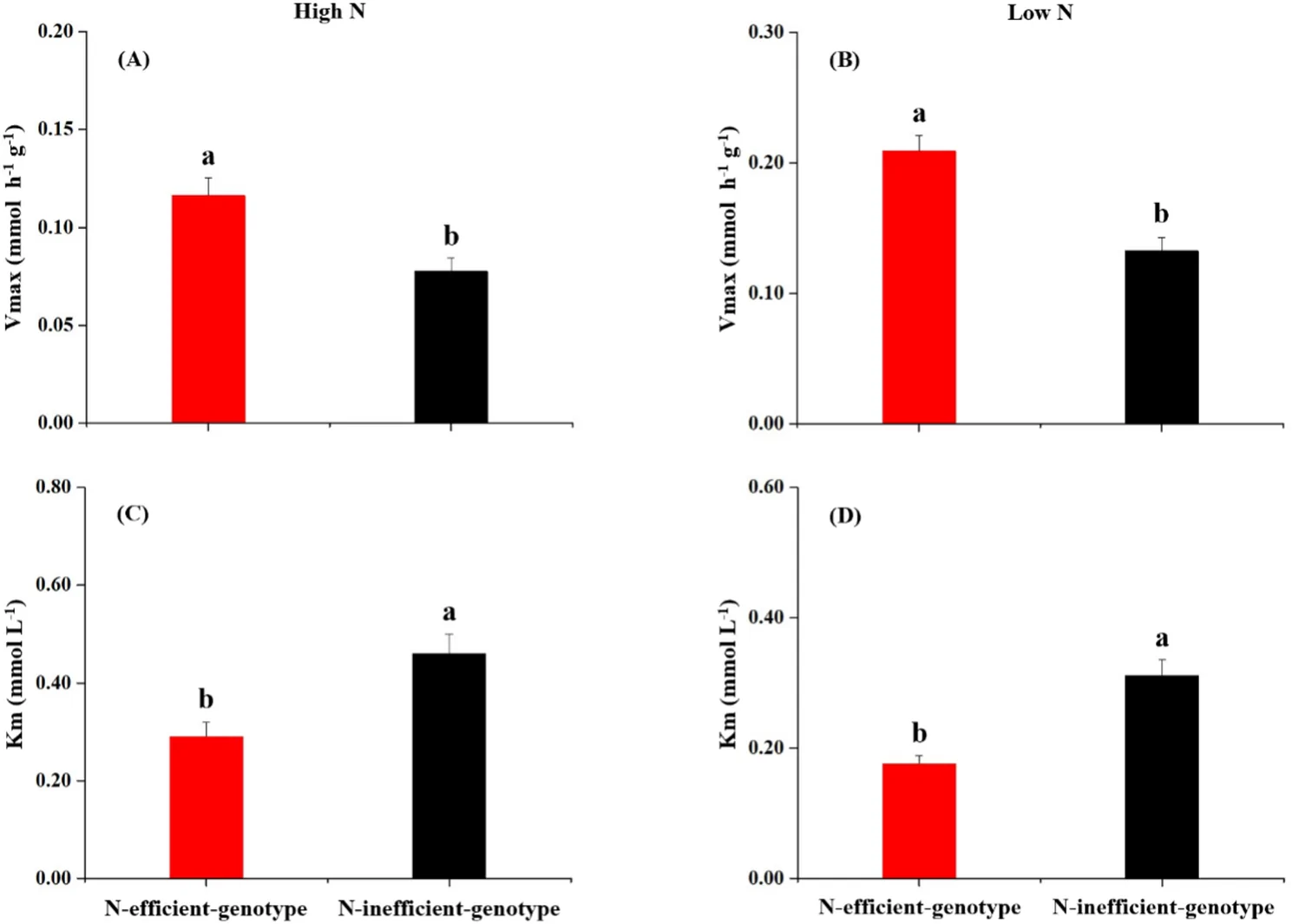
Differences in nitrate uptake kinetic parameters (Vmax (**A**-**B**), the root N kinetic parameter maximum uptake rate; Km (**C**-**D**), the root N kinetic parameter Michaelis constant) between N-efficient-genotype and N-inefficient-genotype under hydroponic culture. Each bar above the mean value represents standard error of the mean (*n* = 3), and different letters above the bars within a figure indicate significant differences according to the ANOVA-protected LSD_0.05_ test

Our results indicated that the N-efficient-genotype exhibited 54.80% higher Vmax than for the N-inefficient-genotype, while the greater the Km of N-inefficient-genotype was 65.69% greater than did the N-efficient-genotype (Fig. 4A-D).

**Fig. 5:**
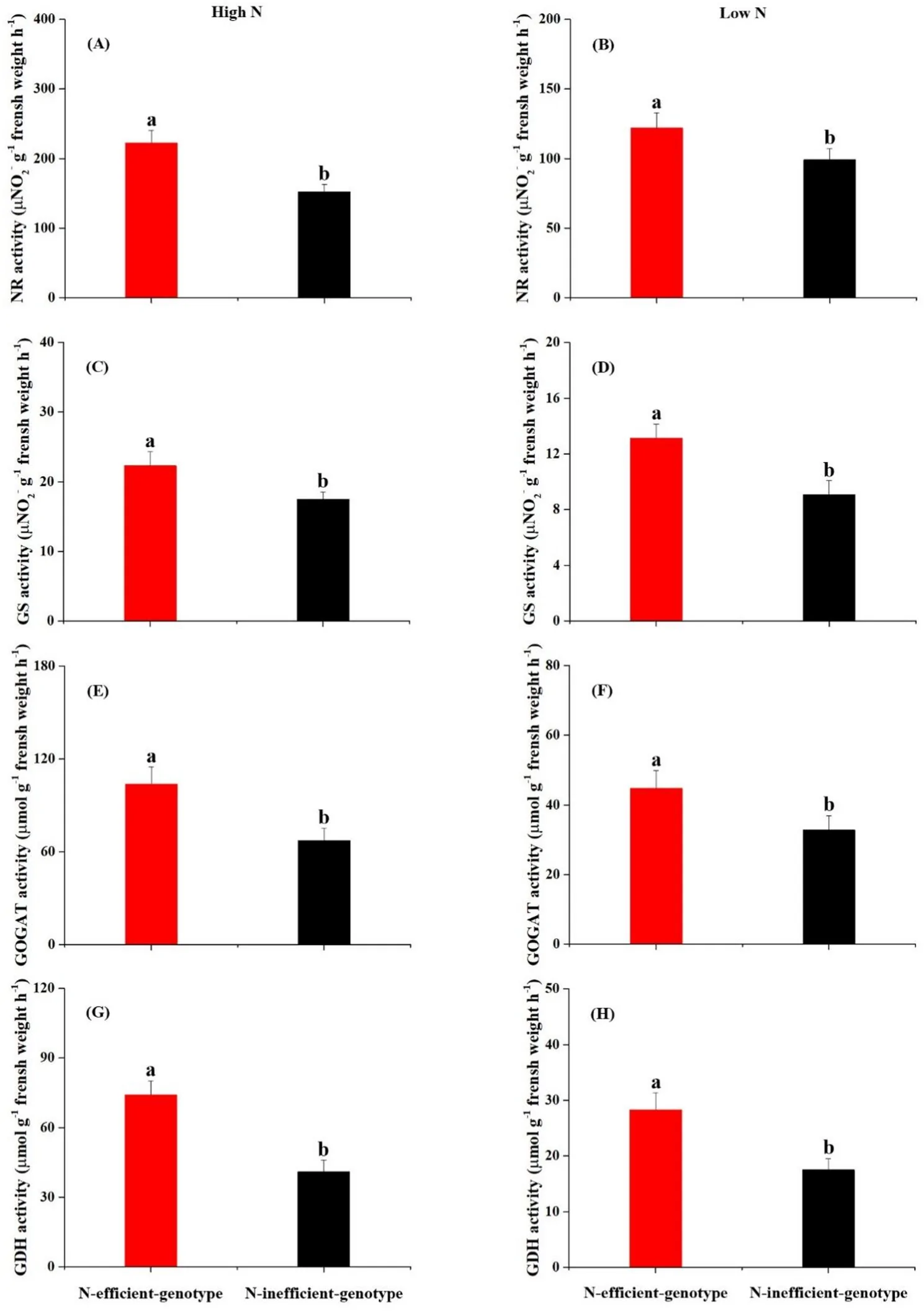
Differences in N metabolism enzyme (NR (**A**-**B**), nitrate reductase; GS (**C**-**D**), glutamine synthetase; GOGAT (**E**-**F**), glutamate synthetase; GDH (**G**-**H**), glutamate dehydrogenase) activity between N-efficient-genotype and N-inefficient-genotype under hydroponic culture. Each bar above the mean value represents standard error of the mean (*n* = 3), and different letters above the bars within a figure indicate significant differences according to the ANOVA-protected LSD_0.05_ test

Overall, it was inferred that the root NR, GS, GOGAT and GDH activity of the N-efficient-genotype were respectively 36.86% (Fig. 5A), 48.68% (Fig. 5B), 33.44% (Fig. 5C) and 75.36% (Fig. 5D) higher than for the N-inefficient-genotype.

**Fig. 6:**
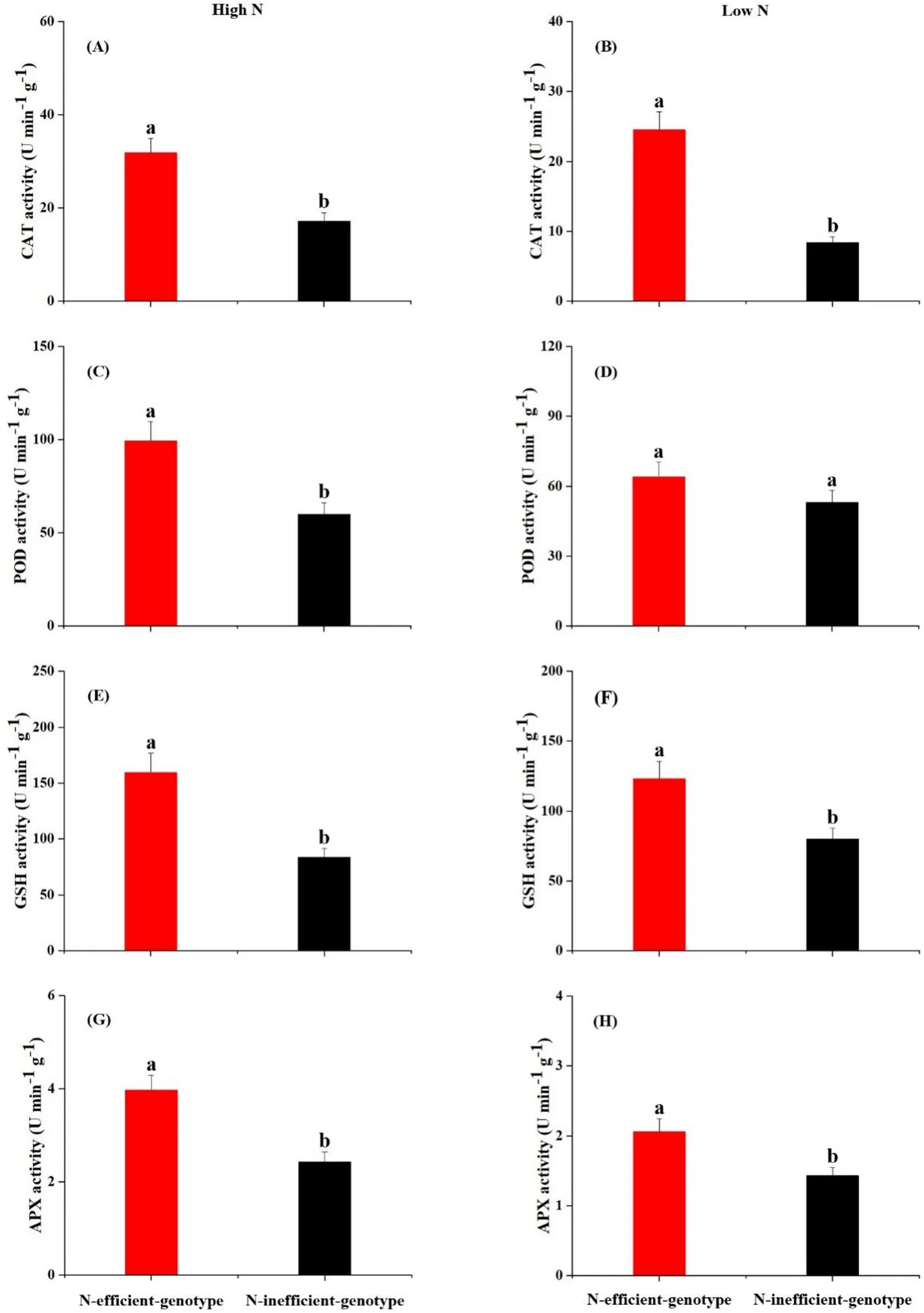
Differences in root senescence-related enzyme (CAT (**A**-**B**), catalase; POD (**C**-**D**), peroxidase; GSH (**E**-**F**), glutathione; APX (**G**-H), ascorbate peroxidase) activity between N-efficient-genotype and N-inefficient-genotype under hydroponic culture. Each bar above the mean value represents standard error of the mean (*n* = 3), and different letters above the bars within a figure indicate significant differences according to the ANOVA-protected LSD_0.05_ test

As expected, the N-efficient-genotype showed 134.14% higher CAT (Fig. 6A-B), 44.52% POD (Fig. 6C-D), 72.71% GSH (Fig. 6E-F), 40.62% APX (Fig. 6G-H), 50.23% SOD activity (Fig. S3A-B) and 43.60% soluble sugar content (Fig. S3C-D) than that for the N-inefficient-genotype.

**Fig. 7:**
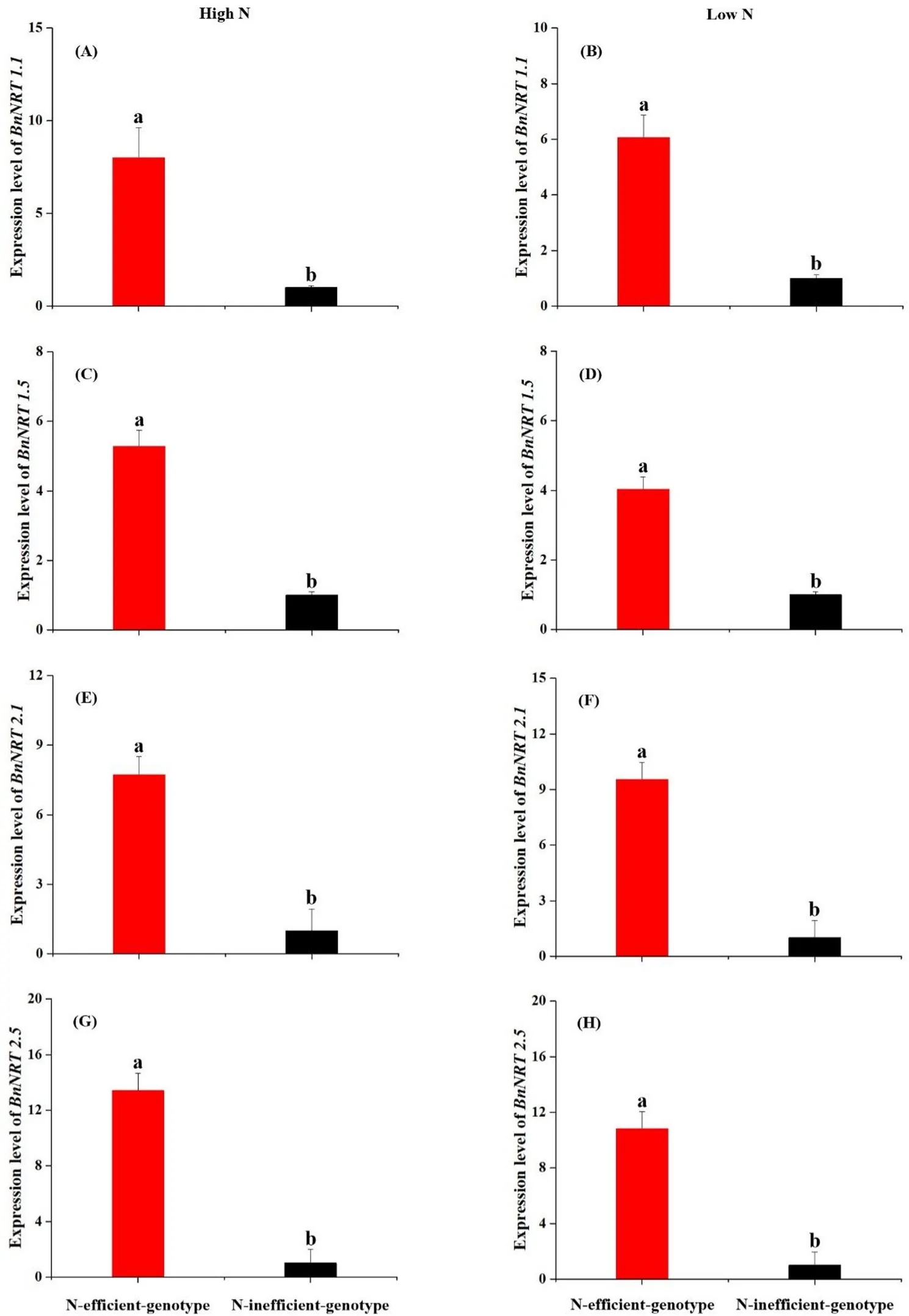
Differences in root relative RNA expression (NRT1, nitrate transporters 1 (*BnNRT1.1*, **A**-**B**; *BnNRT1.5*, **C**-**D**) and NRT2, nitrate transporters 2 (*BnNRT2.1*, E-F; *BnNRT2.5*, **G**-**H**) between N-efficient-genotype and N-inefficient-genotype under hydroponic culture. Each bar above the mean value represents standard error of the mean (*n* = 3), and different letters above the bars within a figure indicate significant differences according to the ANOVA-protected LSD_0.05_ test

**Fig. 8:**
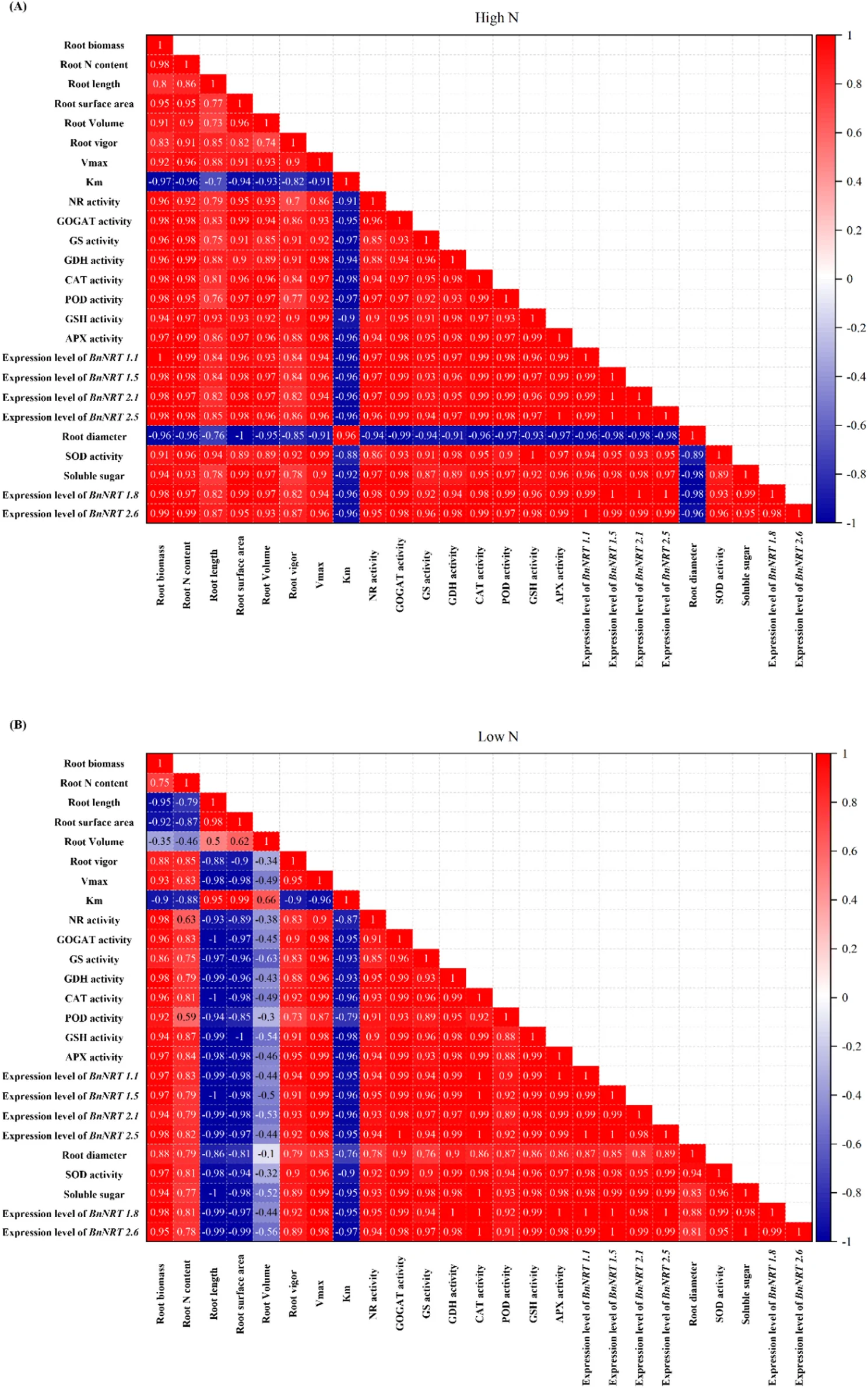
Correlation between all the root traits under the high (**A**) and low (**B**) N levels. A color gradient from blue to red denotes the shift from negative to positive correlation coefficients, with color intensity corresponding to the magnitude of correlation

Root transcription levels were tested, and it was inferred that the RNA expression levels in *BnNRT1.1*, *BnNRT1.5*, *BnNRT1.8*, *BnNRT2.1*, *BnNRT2.5* and *BnNRT2.6* were respectively 7.04 (Fig. 7A-B), 4.66 (Fig. 7C-D), 3.94 (Fig. S4A-B), 8.63 (Fig. 7E-F), 12.13 (Fig. 7G-H) and 7.60 (Fig. S4C-D) fold higher for the N-efficient-genotype than for the N-inefficient-genotype (Fig. 8).

### Correlation between the root traits in the hydroponic culture

Correlation analysis of all root traits confirmed that there was a consistent positive correlation among most of the measured traits under high and low N levels (Fig. 8A-B). Among them, the root biomass exhibited strong positive associations with root length, surface area, and volume. Similarly, we observed a high positive correlation between root senescence-related enzyme activity and root vigor. In addition, root relative RNA expression levels are closely linked to the activity of N metabolism enzymes.

## Discussion

**Fig. 9:**
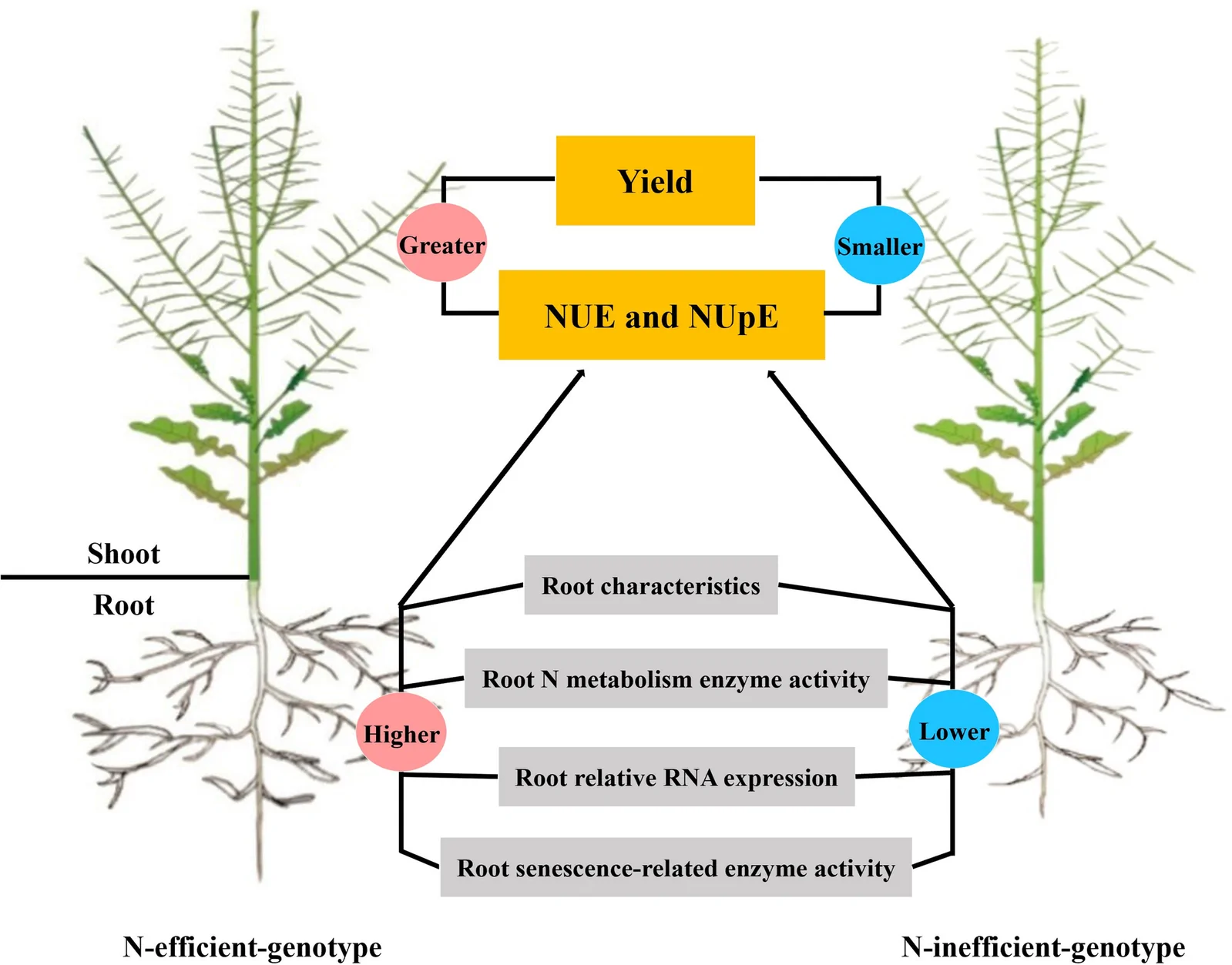
Simplified model for root traits determining NUpE between the N-inefficient-genotype and N-efficient-genotype. Compared to the N-inefficient-genotype, the N-efficient-genotype displayed the slower root senescence-related enzyme activity, higher root relative RNA expression, root N metabolism enzyme activity and root characteristics, leading to the larger leaf photosynthetic parameters, and ultimately achieve the greater NUE, NUpE and yield in rapeseed genotypes

This study showed that regardless of the high and low N environments, the root biomass was the main factor affecting NUpE at the pot experiment (Fig. 1), which validated our hypothesis. Under hydroponic culture, we demonstrated that the N-efficient-genotype displayed significantly higher root biomass and N content (Fig. 2), accompanied by greater root characteristics (Fig. 3), nitrate uptake kinetic parameters (Fig. 4), N metabolism enzyme activity (Fig. 5), root senescence-related enzyme activity (Fig. 6) and root relative RNA expression (Fig. 7), which may lead to the larger N uptake ability than did the N-efficient-genotype at the high and low N levels (Fig. 9).

Given economically and environmentally friendly use of valuable N resources, addressing NUpE as an important component to improve NUE would be necessary, and selection key traits related to NUpE may be achieve this goal [31]. According to the formula of NUpE (shoot N accumulation / N supply), the shoot N accumulation was divided into 10 plant indicators, and the relationship between plant indicators and NUpE was tested. Our results indicated that root biomass was the main factor determining NUpE by principal component, path and regression analysis (Fig. 1 and S1), which was consistent with the previous researches [32–34]. The dominance of root biomass in determining NUpE aligned with the “root economic space” concept [35], which posited that larger root systems invested more carbon in soil exploration, thereby increasing the probability of encountering nitrate patches. This result demonstrated that increasing root biomass could support efficient N uptake from the soil followed by high N metabolism and N use efficiency [36], because root system is an important organ for N uptake and support the healthy growth and development of aboveground parts [37]. At the same time, plant rooting strategy for growth and survival depends on biomass of root, and the root biomass was related plant N uptake [35, 38]. Based on this, the focus of subsequent research would be on plant root, especially on comparing the differences in root physiological indicators with the contrasting NUpE genotypes.

Root is the primary organ in plants that absorb nutrients and water, and root characteristics are associated with the N uptake and has an essential role in N acquisition [39, 40]. Compared to the N-inefficient wheat genotypes (XN509 and AK58), the N-efficient genotypes (YM49 and ZM27) produced the larger root characteristics, which may be responsible for the higher root biomass accumulation and N metabolism to support efficient N uptake followed by greater NUpE and NUE [41]. Similar result was found in our study, which we demonstrated that the N-efficient-genotype exhibited the higher root characteristics (Fig. 3) than did the N-inefficient-genotype, which may result in the greater N uptake ability to promote root oxidation and leaf photosynthesis rate [32, 42]. On the other hand, nitrate uptake kinetics is a useful tool to examine the N uptake rates at a range of N concentrations [43], and the maximum uptake rate (Vmax) and half saturation constant (Km) are used to examine the competitive ability to acquire N, respectively [44]. According to our study, we reported that Vmax of the N-efficient-genotype was greater than that of the N-inefficient-genotype, but N-inefficient-genotype showed the larger Km than did the N-efficient-genotype (Fig. 4). It should be noted that the pot experiment was harvested at maturity (BBCH 89), whereas the hydroponic experiment sampled at the pod-filling stage (BBCH 75). Although root senescence progressed rapidly and N allocation patterns might differ between these developmental stages, root biomass remained the dominant predictor of NUpE across both experimental systems, suggesting that root-driven N uptake capacity would be established by the pod-filling stage largely contributing to final N accumulation. This result is consistent with manipulation experiments [45], suggesting that the affinity of N-efficient-genotype for N uptake is greater than that for N-inefficient-genotype, and higher root N uptake ability is an important physiological mechanism in N-efficient-genotype that enables this cultivar to efficiently acquire N, particularly during the pod-filling stage [46, 47].

It is well known that N metabolism of rapeseed starts from the uptake of NO_3_^−^ by root [42, 48]. After uptake, the NO_3_^−^ is reduced to NH_4_^+^ by NR and nitrite reductase to enter the ammonium assimilation process [24, 49]. Finally, the NH_4_^+^ enters the glutamatecycle and converts into glutamine and glutamate, which are used for the synthesis of amino acids and proteins, by GS and GOGAT as well as by GDH [36, 50]. Our results indicated that N-efficient-genotype exhibited the higher root NR, GS, GOGAT and GDH activity than that of N-inefficient-genotype (Fig. 5), which indicated that greater N uptake potential of N-efficient-genotype might be due to a large-developed root system (Figs. 2, 3 and 4) and higher demand for N in leaf photosynthesis [51]. Our previous research found that compared with the N-inefficient rapeseed genotypes, the N-efficient rapeseed genotypes displayed greater photosynthesis sustained during the post-flowering period, and we speculated this phenomenon was significantly correlated with the N uptake by root system, especially the N metabolism enzyme in root [16]. The N from outside was uptake by rapeseed root, and was mainly assimilated in the shoots for leaf photosynthesis depending on the activity of N metabolism enzyme [52, 53]. Thus, the higher photosynthesis sustained was due to a well-organized system of N uptake system, as described by its greater N metabolism enzyme activity related to N metabolism [54, 55].

Shaping morphological and physiological root traits are important determinants for providing water and nutrients to shoots, and most of these processes undergo age-dependent progression, increasing during early vegetative growth but declining at senescence stage [56, 57]. Senescence represents the final stage of plant development, and reactive oxygen species (ROS) accumulates, accompanied by decreasing in root senescence-related enzyme activity, which plays important role in scavenging excess ROS [58]. At pod-filling stage, root senescence-related enzyme activity (CAT, POD, GSH, APX and SOD; Fig. 6 and Fig. S3A-B) and soluble sugar content (Fig. S3C-D) for the N-efficient-genotype were respectively higher than for the N-inefficient-genotype, indicating N-efficient-genotype could keep the activities of proteins, lipids and chlorophyll, which correlated with maintaining many physiological functions of root cells and delay the root senescence [59]. Rather than simply listing higher activities, we proposed a mechanistic model in which elevated CAT, POD, GSH, APX and SOD activity prevents oxidative damage, thereby delaying root senescence and prolonging N uptake during the pod-filling stage [59, 60]. In a preliminary study, we used ^15^N-labeling technique to verify that compare to N-inefficient rapeseed genotypes, the root of N-efficient rapeseed genotypes absorbed and remobilized the greater N for seed N accumulation [13]. According to this study, we could draw a conclusion that the N-efficient-genotype showed the slower root senescence, which may lead to the stronger and longer N uptake ability, and could absorb more N to support the greater photosynthesis sustained and larger NUE at the pod-filling stage [61, 62].

Plants have evolved sophisticated mechanisms for NO_3_^−^ uptake, which is mainly carried out via transport systems: low-affinity transport system (LATS, concentration of nitrate was higher than 1 mM), high-affinity transport system (HATS, concentration of nitrate was lower than 1 mM) [63, 64]. Nitrate transporter 1 (NRT1, low-affinity transporter; LAT) and NRT2 (high-affinity transporter; HAT) family are two most studied nitrate transporter families in plants [65]. In rapeseed plants, NRT1 gene family and NRT2 gene family were confirmed to be related N uptake, such as *BnNRT1.1*, *BnNRT 1.5*, *BnNRT 1.8*, *BnNRT 2.1*, *BnNRT 2.5* and *BnNRT 2.6* [66, 67]. In this study, our results revealed that greater N uptake capacity for N-efficient-genotype was reflected in higher transcription levels of certain putative transporter genes (*BnNRT1.1*, *BnNRT1.5*, *BnNRT1.8*, *BnNRT2.1*, *BnNRT2.5* and *BnNRT2.6*) (Fig. 7 and S4). This was consistent with previous research findings, which reported that compared to the N-inefficient genotypes, the N-efficient genotypes showed higher the expression levels of *BnNRT1.1* and *BnNRT2.1* in plant root, which was associated with greater N uptake ability to absorb the larger N from the environment [23, 67]. Although the function of these genes could not be verified in this study (e.g., via overexpression or knockout), the consistent upregulation of multiple *BnNRT* genes in N-efficient genotype suggested a coordinated transcriptional program that likely contributes to enhanced N uptake capacity, and future studies employing transgenic approaches would be warranted to confirm these causal relationships. Although whole-root RNA extraction was used in this study, which may have diluted zone-specific signals, such findings could imply that the higher N uptake potential for the N-efficient-genotype has a great influence on dry matter accumulation and translocation, allowing an increased supply of N to rapeseed effective ovule number to promote their development into effective seed, and ultimately improve the NUE for environmentally friendly farming systems [68, 69].

## Conclusion

The rapeseed root was the main factor affecting NUpE, and the N-efficient-genotype displayed greater root biomass, higher Vmax, elevated N‑metabolism enzyme activities, and upregulated NRT expression collectively provided a mechanistic basis for the enhanced N acquisition, which may lead to greater N uptake ability, such that ultimately enhanced NUpE, NUE and yield. Importantly, these differences were evident at the pod‑filling stage, when N demand for seed filling would be high, underscoring the agronomic relevance of root traits during reproductive development. Our results suggest that further study could establish a set of scientific and reasonable root evaluation method to evaluate the N-efficient-genotype in rapeseed, so as to fully tap the N uptake potential of rapeseed and lay a foundation for improving the NUE through genetic approaches for breeding selection. Based on this, we believe that the N-efficient-genotype could be considered as a potential reference genotype to meet the requirement for a more environmentally friendly agriculture and increasing food demand for a predicted world population growth. On the other hand, we recognized that our study was based on only two contrasting rapeseed genotypes (No. 44 and No. 41) selected from a larger panel, and that the absence of lateral root and root hair measurements restricted the resolution of root architectural analysis, which might weaken the practical application of the core conclusion that “root traits may facilitate NUpE”. Therefore, the conclusions drawn here should be regarded as specific to the studied two genotypes and controlled pot or hydroponic conditions. We believed further study could select a sufficient number of rapeseed genotypes from the rapeseed population as samples and supplement the measurements of lateral root and root hair to fully disentangle the root architectural determinants of NUpE in physiological and molecular.

## Supplementary Information

Supplementary Material 1.

## Data Availability

No datasets were generated or analyzed in this study.
