# Supplementary material for: Root traits are strongly associated with nitrogen uptake efficiency in contrasting rapeseed genotypes
Source: BMC Plant Biol. 2026 Aug 5;26:1566. doi: 10.1186/s12870-026-09682-5 (PMC13560114; doi:10.1186/s12870-026-09682-5)
Supplement: Supplementary file 1 [file 12870_2026_9682_MOESM1_ESM.docx]

**Supplementary data**

Table 1 Nitrogen uptake efficiency-related indicators of 50 rapeseed genotypes under high N level (Values are means ± SE (n = 4))

| No. | seed biomass (g) | silique husk biomass (g) | stem biomass (g) | leaf biomass (g) | root biomass (g) | seed N content (%) | Silique husk N content (%) | stem N content (%) | leaf N content (%) | root N content (%) | NUpE |
| --- | --- | --- | --- | --- | --- | --- | --- | --- | --- | --- | --- |
| 1 | 6.05 ± 0. 53 | 6.46 ± 0.51 | 5.58 ± 0.48 | 1.69 ± 0.08 | 2.41 ± 0.15 | 5.30 ± 0.38 | 2.48 ± 0.18 | 1.42 ± 0.08 | 0.82 ± 0.05 | 1.19 ± 0.10 | 0.38 ± 0.02 |
| 2 | 7.02 ± 0.64 | 7.63 ± 0.64 | 3.52 ± 0.27 | 1.20 ± 0.07 | 2.43 ± 0.14 | 4.91 ± 0.31 | 2.29 ± 0.15 | 0.96 ± 0.05 | 0.86 ± 0.05 | 0.91 ± 0.08 | 0.34 ± 0.02 |
| 3 | 5.32 ± 0.46 | 6.24 ± 0.48 | 3.78 ± 0.30 | 1.46 ± 0.07 | 1.08 ± 0.09 | 4.58 ± 0.30 | 2.41 ± 0.16 | 1.03 ± 0.0.9 | 0.56 ± 0.03 | 1.09 ± 0.09 | 0.29 ± 0.01 |
| 4 | 4.07 ± 0.35 | 5.69 ± 0.43 | 2.54 ± 0.21 | 1.19 ± 0.06 | 1.26 ± 0.11 | 4.45 ± 0.31 | 2.20 ± 0.15 | 1.74 ± 0.09 | 0.61 ± 0.03 | 1.12 ± 0.10 | 0.27 ± 0.01 |
| 5 | 6.39 ± 0.59 | 8.39 ± 0.79 | 5.19 ± 0.48 | 1.50 ± 0.07 | 1.97 ± 0.12 | 4.88 ± 0.33 | 2.30 ± 0.14 | 1.34 ± 0.07 | 0.90 ± 0.05 | 0.97 ± 0.08 | 0.38 ± 0.02 |
| 6 | 6.21 ± 0.51 | 5.97 ± 0.43 | 3.93 ± 0.31 | 1.44 ± 0.07 | 1.30 ± 0.10 | 4.52 ± 0.34 | 2.39 ± 0.18 | 2.28 ± 0.12 | 0.59 ± 0.04 | 1.29 ± 0.10 | 0.38 ± 0.02 |
| 7 | 4.61 ± 0.35 | 4.31 ± 0.31 | 1.63 ± 0.10 | 1.07 ± .005 | 0.52 ± 0.01 | 4.31 ± 0.37 | 2.31 ± 0.16 | 1.32 ± 0.07 | 0.46 ± 0.02 | 1.42 ± 0.11 | 0.19 ± 0.01 |
| 8 | 5.75 ± 0.49 | 7.92 ± 0.64 | 1.90 ± 0.11 | 0.84 ± 0.01 | 0.81 ± 0.01 | 4.22 ± 0.35 | 2.15 ± 0.15 | 1.22 ± 0.07 | 0.42 ± 0.03 | 1.23 ± 0.12 | 0.29 ± 0.02 |
| 9 | 8.16 ± 0.69 | 7.62 ± 0.61 | 3.49 ± 0.29 | 1.16 ± 0.06 | 1.45 ± 0.09 | 4.77 ± 0.38 | 1.94 ± 0.10 | 2.11 ± 0.11 | 0.69 ± 0.05 | 1.64 ± 0.13 | 0.39 ± 0.02 |
| 10 | 5.70 ± 0.42 | 6.56 ± 0.51 | 2.94 ± 0.20 | 1.16 ± 0.06 | 2.29 ± 0.17 | 5.09 ± 0.40 | 2.33 ± 0.13 | 1.04 ± 0.06 | 0.65 ± 0.04 | 1.17 ± 0.0.9 | 0.33 ± 0.02 |
| 11 | 3.83 ± 0.31 | 4.34 ± 0.35 | 2.63 ± 0.19 | 1.43 ± 0.07 | 1.21 ± 0.08 | 5.09 ± 0.41 | 2.48 ± 0.16 | 2.21 ± 0.18 | 0.40 ± 0.03 | 1.19 ± 0.08 | 0.29 ± 0.02 |
| 12 | 9.13 ± 0.84 | 11.78 ± 1.09 | 8.14 ± 0.75 | 1.54 ± 0.07 | 4.52 ± 0.28 | 4.51 ± 0.38 | 2.73 ± 0.16 | 1.36 ± 0.08 | 0.81 ± 0.06 | 1.04 ± 0.08 | 0.58 ± 0.04 |
| 13 | 10.72 ± 0.89 | 7.13 ± 0.59 | 10.30 ± 0.91 | 2.03 ± 0.15 | 7.24 ± 0.61 | 5.67 ± 0.45 | 2.22 ± 0.15 | 1.06 ± 0.06 | 1.19 ± 0.09 | 1.13 ± 0.08 | 0.58 ± 0.04 |
| 14 | 6.76 ± 0.57 | 10.06 ± 0.92 | 7.17 ± 0.62 | 1.63 ± 0.08 | 4.45 ± 0.32 | 4.24 ± 0.31 | 1.96 ± 0.09 | 1.19 ± 0.07 | 0.81 ± 0.06 | 1.35 ± 0.11 | 0.44 ± 0.03 |
| 15 | 8.47 ± 0.71 | 8.71 ± 0.65 | 5.84 ± 0.49 | 1.47 ± 0.07 | 2.91 ± 0.19 | 4.63 ± 0.35 | 2.53 ± 0.15 | 1.16 ± 0.07 | 0.38 ± 0.02 | 0.96 ± 0.07 | 0.35 ± 0.02 |
| 16 | 6.27 ± 0.52 | 5.23 ± 0.41 | 3.83 ± 0.28 | 1.54 ± 0.06 | 1.52 ± 0.09 | 5.03 ± 0.38 | 2.05 ± 0.12 | 1.95 ± 0.11 | 0.77 ± 0.06 | 1.40 ± 0.11 | 0.35 ± 0.02 |
| 17 | 8.54 ± 0.71 | 11.51 ± 1.01 | 5.54 ± 0.48 | 1.27 ± 0.05 | 3.72 ± 0.28 | 5.31 ± 0.42 | 2.79 ± 0.19 | 0.96 ± 0.06 | 0.92 ± 0.07 | 1.05 ± 0.08 | 0.54 ± 0.03 |
| 18 | 5.16 ± 0.40 | 4.98 ± 0.32 | 1.76 ± 0.11 | 1.00 ± 0.01 | 0.87 ± 0.01 | 4.80 ± 0.35 | 2.29 ± 0.14 | 1.22 ± 0.08 | 0.22 ± 0.01 | 1.31 ± 0.10 | 0.24 ± 0.02 |
| 19 | 5.78 ± 0.48 | 6.94 ± 0.51 | 4.63 ± 0.35 | 1.54 ± 0.06 | 1.73 ± 0.09 | 5.00 ± 0.36 | 2.40 ± 0.12 | 1.88 ± 0.10 | 0.50 ± 0.03 | 1.86 ± 0.15 | 0.40 ± 0.03 |
| 20 | 7.52 ± 0.62 | 7.94 ± 0.63 | 4.07 ± 0.32 | 1.29 ± 0.04 | 2.71 ± 0.15 | 4.89 ± 0.34 | 1.80 ± 0.09 | 1.73 ± 0.09 | 0.52 ± 0.04 | 1.37 ± 0.11 | 0.46 ± 0.03 |
| 21 | 6.70 ± 0.56 | 11.27 ± 1.08 | 6.20 ± 0.55 | 1.47 ± 0.07 | 2.32 ± 0.11 | 4.69 ± 0.34 | 2.21 ± 0.14 | 0.99 ± 0.05 | 0.66 ± 0.03 | 1.44 ± 0.12 | 0.48 ± 0.03 |
| 22 | 6.59 ± 0.51 | 10.83 ± 0.95 | 5.52 ± 0.48 | 1.37 ± 0.07 | 3.34 ± 0.19 | 4.83 ± 0.32 | 2.27 ± 0.15 | 1.24 ± 0.09 | 0.78 ± 0.06 | 1.09 ± 0.08 | 0.47 ± 0.03 |
| 23 | 10.21 ± 0.90 | 10.58 ± 0.92 | 6.35 ± 0.56 | 1.30 ± 0.06 | 3.60 ± 0.28 | 4.88 ± 0.33 | 2.29 ± 0.15 | 1.13 ± 0.08 | 1.20 ± 0.09 | 1.21 ± 0.10 | 0.57 ± 0.04 |
| 24 | 7.96 ± 0.68 | 11.52 ± 1.06 | 7.16 ± 0.62 | 1.46 ± 0.07 | 4.12 ± 0.33 | 4.53 ± 0.30 | 2.55 ± 0.16 | 1.06 ± 0.06 | 0.92 ± 0.07 | 1.10 ± 0.09 | 0.51 ± 0.04 |
| 25 | 6.22 ± 0.56 | 9.57 ± 0.81 | 6.51 ± 0.58 | 1.91 ± 0.11 | 6.96 ± 0.52 | 5.05 ± 0.41 | 2.27 ± 0.15 | 1.93 ± 0.12 | 1.00 ± 0.08 | 1.06 ± 0.07 | 0.63 ± 0.05 |
| 26 | 10.57 ± 0.92 | 9.57 ± 0.83 | 9.60 ± 0.83 | 1.69 ± 0.08 | 11.50 ± 1.08 | 4.68 ± 0.35 | 2.75 ± 0.19 | 1.52 ± 0.09 | 1.46 ± 0.11 | 1.48 ± 0.11 | 0.77 ± 0.05 |
| 27 | 6.75 ± 0.53 | 8.78 ± 0.71 | 9.42 ± 0.88 | 1.83 ± 0.09 | 6.94 ± 0.55 | 5.07 ± 0.40 | 2.63 ± 0.17 | 1.22 ± 0.07 | 1.11 ± 0.09 | 1.88 ± 0.14 | 0.59 ± 0.04 |
| 28 | 8.05 ± 0.69 | 6.61 ± 0.55 | 3.88 ± 0.30 | 1.20 ± 0.05 | 4.84 ± 0.32 | 4.54 ± 0.32 | 2.74 ± 0.19 | 1.25 ± 0.07 | 0.67 ± 0.04 | 1.42 ± 0.11 | 0.43 ± 0.03 |
| 29 | 9.37 ± 0.81 | 9.27 ± 0.78 | 4.68 ± 0.38 | 1.22 ± 0.05 | 3.17 ± 0.21 | 4.83 ± 0.34 | 2.43 ± 0.15 | 1.08 ± 0.07 | 0.50 ± 0.04 | 1.59 ± 0.13 | 0.48 ± 0.03 |
| 30 | 6.24 ± 0.53 | 6.43 ± 0.56 | 3.26 ± 0.27 | 1.25 ± 0.05 | 1.20 ± 0.08 | 4.88 ± 0.32 | 2.00 ± 0.11 | 1.26 ± 0.08 | 0.34 ± 0.02 | 1.49 ± 0.12 | 0.38 ± 0.03 |
| 31 | 8.36 ± 0.79 | 8.32 ± 0.72 | 3.83 ± 0.30 | 1.18 ± 0.04 | 1.92 ± 0.11 | 4.44 ± 0.32 | 2.03 ± 0.13 | 1.30 ± 0.07 | 0.74 ± 0.06 | 1.22 ± 0.10 | 0.36 ± 0.02 |
| 32 | 7.86 ± 0.64 | 8.00 ± 0.68 | 6.57 ± 0.52 | 1.67 ± 0.08 | 2.14 ± 0.12 | 4.60 ± 0.34 | 2.08 ± 0.12 | 1.02 ± 0.05 | 0.97 ± 0.07 | 1.16 ±0.10 | 0.46 ± 0.03 |
| 33 | 4.37 ± 0.32 | 9.14 ± 0.88 | 3.36 ± 0.24 | 1.21 ± 0.06 | 1.70 ± 0.09 | 4.35 ± 0.33 | 2.51 ± 0.13 | 1.43 ± 0.11 | 0.37 ± 0.02 | 1.55 ± 0.12 | 0.31 ± 0.02 |
| 34 | 10.57 ± 0.93 | 8.91 ± 0.71 | 5.21 ± 0.46 | 1.20 ± 0.05 | 4.84 ± 0.33 | 3.97 ± 0.28 | 1.73 ± 0.10 | 1.33 ± 0.10 | 0.81 ± 0.06 | 1.36 ± 0.11 | 0.48 ± 0.03 |
| 35 | 10.20 ± 0.87 | 6.89 ± 0.53 | 8.82 ± 0.74 | 1.69 ± 0.09 | 5.30 ± 0.34 | 4.55 ± 0.33 | 2.34 ± 0.15 | 1.59 ± 0.11 | 0.61 ± 0.05 | 0.94 ± 0.07 | 0.51 ± 0.03 |
| 36 | 6.69 ± 0.55 | 7.16 ± 0.55 | 4.09 ± 0.29 | 1.40 ± 0.08 | 1.63 ± 0.09 | 4.91 ± 0.38 | 2.45 ± 0.18 | 1.32 ± 0.11 | 0.68 ± 0.05 | 1.41 ± 0.11 | 0.38 ± 0.02 |
| 37 | 11.91 ± 1.02 | 9.85 ± 0.81 | 4.16 ± 0.31 | 1.00 ± 0.05 | 3.31 ± 0.18 | 4.13 ± 0.32 | 2.54 ± 0.18 | 1.47 ± 0.12 | 1.07 ± 0.08 | 1.23 ± 0.12 | 0.53 ± 0.04 |
| 38 | 8.31 ± 0.71 | 7.50 ± 0.61 | 6.08 ± 0.49 | 1.49 ± 0.08 | 2.60 ± 0.19 | 4.93 ± 0.37 | 2.57 ± 0.17 | 1.43 ± 0.12 | 0.40 ± 0.03 | 1.65 ± 0.13 | 0.46 ± 0.03 |
| 39 | 6.00 ± 0.48 | 8.48 ± 0.75 | 4.01 ± 0.28 | 1.22 ± 0.08 | 4.12 ± 0.35 | 5.29 ± 0.42 | 2.28 ± 0.16 | 0.91 ± 0.08 | 0.54 ± 0.04 | 1.20 ± 0.10 | 0.38 ± 0.02 |
| 40 | 5.87 ± 0.45 | 5.02 ± 0.37 | 2.07 ± 0.11 | 1.03 ± 0.06 | 1.18 ± 0.09 | 4.49 ± 0.39 | 2.04 ± 0.14 | 0.97 ± 0.08 | 0.33 ± 0.02 | 1.63 ± 0.14 | 0.27 ± 0.01 |
| 41 | 3.30 ± 0.25 | 5.36 ± 0.39 | 3.37 ± 0.29 | 1.60 ± 0.09 | 1.31 ± 0.11 | 4.28 ± 0.35 | 3.10 ± 0.21 | 1.02 ± 0.08 | 0.47 ± 0.03 | 1.19 ± 0.09 | 0.23 ± 0.01 |
| 42 | 9.17 ± 0.80 | 10.33 ± 0.92 | 7.19 ± 0.62 | 1.59 ± 0.08 | 2.33 ± 0.19 | 4.64 ± 0.34 | 2.60 ± 0.19 | 1.38 ± 0.09 | 0.96 ± 0.07 | 1.15 ± 0.10 | 0.56 ± 0.04 |
| 43 | 6.40 ± 0.51 | 9.35 ± 0.81 | 5.69 ± 0.42 | 1.53 ± 0.08 | 2.65 ± 0.20 | 4.95 ± 0.38 | 2.64 ± 0.19 | 1.38 ± 0.10 | 0.85 ± 0.06 | 1.10 ± 0.08 | 0.47 ± 0.03 |
| 44 | 7.66 ± 0.62 | 8.84 ± 0.68 | 8.50 ± 0.71 | 1.94 ± 0.09 | 4.06 ± 0.32 | 4.49 ± 0.38 | 3.45 ± 0.28 | 1.51 ± 0.12 | 1.18 ± 0.09 | 1.56 ± 0.13 | 0.72 ± 0.05 |
| 45 | 8.84 ± 0.70 | 8.68 ± 0.68 | 6.06 ± 0.49 | 1.50 ± 0.08 | 2.59 ± 0.21 | 4.46 ± 0.38 | 2.78 ± 0.22 | 1.13 ± 0.09 | 0.71 ± 0.05 | 0.99 ± 0.07 | 0.49 ± 0.03 |
| 46 | 9.25 ± 0.79 | 8.47 ± 0.65 | 7.11 ± 0.61 | 1.59 ± 0.08 | 3.71 ± 0.29 | 4.55 ± 0.37 | 2.28 ± 0.16 | 1.32 ± 0.11 | 0.84 ± 0.06 | 1.57 ± 0.14 | 0.50 ± 0.03 |
| 47 | 3.34 ± 0.21 | 6.44 ± 0.55 | 4.39 ± 0.30 | 1.62 ± 0.07 | 2.24 ± 0.15 | 4.36 ± 0.38 | 2.82 ± 0.17 | 0.85 ± 0.06 | 0.53 ± 0.03 | 1.41 ± 0.14 | 0.31 ± 0.02 |
| 48 | 8.27 ± 0.71 | 6.02 ± 0.47 | 4.85 ± 0.28 | 1.41 ± 0.09 | 3.13 ± 0.26 | 4.48 ± 0.39 | 2.06 ± 0.12 | 1.22 ± 0.11 | 0.73 ± 0.06 | 1.56 ± 0.13 | 0.42 ± 0.02 |
| 49 | 9.38 ± 0.78 | 7.20 ± 0.67 | 5.04 ± 0.42 | 1.36 ± 0.07 | 2.83 ± 0.19 | 4.40 ± 0.39 | 2.34 ± 0.15 | 0.84 ± 0.06 | 0.67 ± 0.04 | 1.00 ± 0.07 | 0.42 ± 0.02 |
| 50 | 4.66 ± 0.33 | 7.28 ± 0.67 | 5.75 ± 0.49 | 1.78 ± 0.09 | 2.13 ± 0.15 | 5.24 ± 0.45 | 2.69 ± 0.19 | 1.39 ± 0.11 | 0.61 ± 0.04 | 1.07 ± 0.08 | 0.38 ± 0.02 |

Table 2 Nitrogen uptake efficiency-related indicators of 50 rapeseed genotypes under low N level (Values are means ± SE (n = 4))

| No. | seed biomass (g) | silique husk biomass (g) | stem biomass (g) | leaf biomass (g) | root biomass (g) | seed N content (%) | Silique husk N content (%) | stem N content (%) | leaf N content (%) | root N content (%) | NUpE |
| --- | --- | --- | --- | --- | --- | --- | --- | --- | --- | --- | --- |
| 1 | 4.93 ± 0.38 | 4.39 ± 0.31 | 3.74 ± 0.25 | 1.12 ± 0.07 | 1.83 ± 0.11 | 6.35 ± 0.51 | 4.07 ± 0.38 | 1.03 ± 0.07 | 0.73 ± 0.05 | 0.93 ± 0.05 | 0.84 ± 0.04 |
| 2 | 5.12 ± 0.42 | 4.04 ± 0.28 | 2.72 ± 0.18 | 0.88 ± 0.04 | 1.90 ± 0.11 | 5.69 ± 0.45 | 4.10 ± 0.37 | 0.96 ± 0.05 | 0.51 ± 0.03 | 1.07 ± 0.05 | 0.81 ± 0.04 |
| 3 | 5.51 ± 0.45 | 4.22 ± 0.30 | 3.19 ± 0.21 | 0.97 ± 0.05 | 1.66 ± 0.09 | 5.54 ± 0.45 | 4.02 ± 0.38 | 0.93 ± 0.05 | 0.33 ± 0.02 | 0.90 ± 0.06 | 0.79 ± 0.04 |
| 4 | 1.67 ± 0.11 | 1.06 ± 0.07 | 1.00 ± 0.06 | 1.07 ± 0.06 | 0.58 ± 0.03 | 5.24 ± 0.41 | 5.53 ± 0.41 | 2.03 ± 0.12 | 0.23 ± 0.01 | 1.28 ± 0.08 | 0.45 ± 0.02 |
| 5 | 6.07 ± 0.52 | 3.07 ± 0.22 | 2.67 ± 0.18 | 0.90 ± 0.04 | 2.69 ± 0.19 | 5.06 ± 0.38 | 3.24 ± 0.20 | 0.97 ± 0.04 | 0.51 ± 0.03 | 0.92 ± 0.05 | 0.81 ± 0.03 |
| 6 | 5.38 ± 0.48 | 2.48 ± 0.18 | 1.75 ± 0.10 | 0.82 ± 0.04 | 1.14 ± 0.09 | 5.06 ± 0.39 | 4.20 ± 0.38 | 1.90 ± 0.11 | 0.43 ± 0.03 | 1.34 ± 0.09 | 0.79 ± 0.04 |
| 7 | 6.02 ± 0.51 | 2.06 ± 0.12 | 2.38 ± 0.16 | 0.97 ± 0.04 | 1.31 ± 0.10 | 5.31 ± 0.38 | 4.44 ± 0.34 | 1.18 ± 0.09 | 0.29 ± 0.02 | 1.17 ± 0.08 | 0.69 ± 0.03 |
| 8 | 4.59 ± 0.39 | 3.37 ± 0.27 | 2.13 ± 0.15 | 0.82 ± 0.05 | 1.66 ± 0.11 | 5.14 ± 0.40 | 4.83 ± 0.36 | 1.53 ± 0.10 | 0.42 ± 0.03 | 1.04 ± 0.06 | 0.79 ± 0.04 |
| 9 | 6.70 ± 0.60 | 3.22 ± 0.23 | 2.39 ± 0.17 | 0.81 ± 0.05 | 1.61 ± 0.11 | 5.69 ± 0.45 | 3.66 ± 0.28 | 1.46 ± 0.10 | 0.53 ± 0.03 | 1.58 ± 0.10 | 0.89 ± 0.05 |
| 10 | 4.65 ± 0.38 | 3.00 ± 0.22 | 2.65 ± 0.19 | 1.01 ± 0.08 | 1.59 ± 0.10 | 6.03 ± 0.48 | 3.96 ± 0.27 | 0.98 ± 0.06 | 0.49 ± 0.03 | 1.09 ± 0.07 | 0.85 ± 0.04 |
| 11 | 2.43 ± 0.19 | 1.62 ± 0.10 | 1.18 ± 0.07 | 0.87 ± 0.05 | 0.74 ± 0.04 | 5.13 ± 0.40 | 4.45 ± 0.38 | 2.38 ± 0.15 | 0.41 ± 0.02 | 1.47 ± 0.11 | 0.64 ± 0.03 |
| 12 | 7.86 ± 0.66 | 3.47 ± 0.25 | 2.64 ± 0.18 | 0.79 ± 0.04 | 2.40 ± 0.15 | 5.69 ± 0.46 | 3.44 ± 0.21 | 0.74 ± 0.04 | 1.02 ± 0.06 | 0.92 ± 0.06 | 1.10 ± 0.07 |
| 13 | 4.02 ± 0.30 | 3.65 ± 0.26 | 7.03 ± 0.58 | 1.51 ± 0.10 | 5.94 ± 0.41 | 5.98 ± 0.48 | 3.62 ± 0.25 | 0.97 ± 0.06 | 0.64 ± 0.03 | 0.97 ± 0.06 | 1.07 ± 0.06 |
| 14 | 7.55 ± 0.68 | 3.91 ± 0.29 | 3.53 ± 0.24 | 0.94 ± 0.06 | 2.87 ± 0.18 | 4.50 ± 0.42 | 2.21 ± 0.15 | 0.92 ± 0.05 | 0.69 ± 0.03 | 0.95 ± 0.05 | 0.94 ± 0.06 |
| 15 | 7.12 ± 0.62 | 3.50 ± 0.24 | 2.95 ± 0.18 | 0.87 ± 0.05 | 2.77 ± 0.18 | 5.24 ± 0.40 | 2.28 ± 0.18 | 1.13 ± 0.09 | 0.80 ± 0.04 | 1.03 ± 0.05 | 0.96 ± 0.06 |
| 16 | 7.64 ± 0.65 | 3.13 ± 0.18 | 3.02 ± 0.20 | 0.92 ± 0.04 | 1.86 ± 0.10 | 5.36 ± 0.41 | 3.89 ± 0.29 | 1.14 ± 0.10 | 0.44 ± 0.02 | 1.29 ± 0.08 | 1.03 ± 0.05 |
| 17 | 6.60 ± 0.55 | 3.77 ± 0.28 | 2.08 ± 0.11 | 0.68 ± 0.04 | 2.14 ± 0.12 | 5.41 ± 0.42 | 3.49 ± 0.27 | 0.84 ± 0.06 | 1.03 ± 0.07 | 0.70 ± 0.04 | 0.98 ± 0.04 |
| 18 | 3.66 ± 0.28 | 2.09 ± 0.15 | 1.77 ± 0.09 | 1.09 ± 0.07 | 0.92 ± 0.05 | 5.95 ± 0.46 | 4.55 ± 0.35 | 1.43 ± 0.10 | 0.50 ± 0.03 | 1.19 ± 0.08 | 0.61 ± 0.03 |
| 19 | 5.87 ± 0.49 | 3.36 ± 0.24 | 2.16 ± 0.13 | 0.79 ± 0.04 | 1.60 ± 0.10 | 5.52 ± 0.43 | 3.00 ± 0.19 | 1.07 ± 0.07 | 0.68 ± 0.03 | 1.95 ± 0.12 | 0.98 ± 0.06 |
| 20 | 6.39 ± 0.55 | 3.29 ± 0.22 | 2.25 ± 0.15 | 0.76 ± 0.04 | 2.11 ± 0.14 | 5.17 ± 0.39 | 2.29 ± 0.16 | 1.01 ± 0.07 | 0.84 ± 0.04 | 1.22 ± 0.11 | 0.95 ± 0.06 |
| 21 | 6.80 ± 0.56 | 3.04 ± 0.22 | 2.58 ± 0.15 | 0.89 ± 0.05 | 1.20 ± 0.09 | 4.76 ± 0.35 | 2.38 ± 0.15 | 0.85 ± 0.05 | 0.57 ± 0.03 | 1.12 ± 0.08 | 0.88 ± 0.05 |
| 22 | 6.56 ± 0.55 | 3.92 ± 0.30 | 2.61 ± 0.14 | 0.80 ± 0.05 | 2.76 ± 0.15 | 5.04 ± 0.37 | 2.62 ± 0.18 | 0.86 ± 0.04 | 0.65 ± 0.03 | 0.91 ± 0.05 | 0.99 ± 0.05 |
| 23 | 8.11 ± 0.72 | 2.77 ± 0.17 | 3.27 ± 0.21 | 0.87 ± 0.04 | 5.37 ± 0.40 | 4.25 ± 0.32 | 2.29 ± 0.16 | 0.84 ± 0.05 | 0.64 ± 0.03 | 1.52 ± 0.12 | 1.11 ± 0.08 |
| 24 | 7.14 ± 0.63 | 3.74 ± 0.24 | 2.97 ± 0.19 | 0.86 ± 0.05 | 2.63 ± 0.14 | 4.88 ± 0.34 | 3.00 ± 0.19 | 0.84 ± 0.04 | 0.87 ± 0.04 | 0.96 ± 0.05 | 0.94 ± 0.05 |
| 25 | 7.84 ± 0.68 | 2.78 ± 0.18 | 3.68 ± 0.24 | 0.97 ± 0.06 | 4.61 ± 0.28 | 4.64 ± 0.33 | 2.05 ± 0.12 | 1.05 ± 0.06 | 0.97 ± 0.05 | 0.76 ± 0.04 | 0.98 ± 0.06 |
| 26 | 6.59 ± 0.55 | 2.53 ± 0.16 | 4.21 ± 0.31 | 1.02 ± 0.08 | 8.63 ± 0.70 | 4.68 ± 0.32 | 1.68 ± 0.09 | 0.98 ± 0.07 | 1.20 ± 0.09 | 1.28 ± 0.09 | 1.11 ± 0.07 |
| 27 | 8.13 ± 0.68 | 3.25 ± 0.21 | 5.57 ± 0.45 | 1.16 ± 0.09 | 5.35 ± 0.45 | 4.85 ± 0.35 | 2.56 ± 0.18 | 0.65 ± 0.04 | 0.87 ± 0.04 | 1.60 ± 0.10 | 1.15 ± 0.08 |
| 28 | 5.84 ± 0.45 | 2.49 ± 0.25 | 2.08 ± 0.10 | 0.84 ± 0.05 | 1.58 ± 0.08 | 5.06 ± 0.37 | 3.82 ± 0.25 | 1.04 ± 0.08 | 0.83 ± 0.04 | 1.39 ± 0.09 | 0.93 ± 0.06 |
| 29 | 7.63 ± 0.62 | 3.08 ± 0.28 | 3.78 ± 0.28 | 1.09 ± 0.08 | 2.10 ± 0.11 | 5.17 ± 0.37 | 2.18 ± 0.14 | 0.88 ± 0.04 | 0.90 ± 0.05 | 1.15 ± 0.08 | 0.94 ± 0.06 |
| 30 | 5.72 ± 0.44 | 2.96 ± 0.22 | 3.29 ± 0.22 | 1.11 ± 0.09 | 1.44 ± 0.07 | 5.04 ± 0.36 | 2.82 ± 0.19 | 0.95 ± 0.06 | 0.88 ± 0.04 | 1.08 ± 0.06 | 0.91 ± 0.05 |
| 31 | 8.04 ± 0.68 | 3.72 ± 0.25 | 3.61 ± 0.22 | 0.97 ± 0.05 | 2.06 ± 0.11 | 4.87 ± 0.38 | 2.72 ± 0.17 | 0.76 ± 0.05 | 0.51 ± 0.03 | 0.98 ± 0.06 | 0.93 ± 0.05 |
| 32 | 6.92 ± 0.53 | 3.70 ± 0.26 | 3.24 ± 0.23 | 0.94 ± 0.05 | 2.28 ± 0.15 | 4.82 ± 0.30 | 2.43 ± 0.15 | 0.93 ± 0.07 | 0.68 ± 0.04 | 0.85 ± 0.05 | 0.92 ± 0.05 |
| 33 | 2.88 ± 0.19 | 5.27 ± 0.41 | 3.54 ± 0.25 | 1.03 ± 0.07 | 2.53 ± 0.14 | 5.20 ± 0.38 | 4.38 ± 0.325 | 1.33 ± 0.10 | 0.64 ± 0.03 | 1.48 ± 0.10 | 0.83 ± 0.05 |
| 34 | 7.75 ± 0.66 | 3.64 ± 0.26 | 2.99 ± 0.18 | 0.79 ± 0.04 | 4.64 ± 0.32 | 4.20 ± 0.35 | 2.08 ± 0.11 | 0.95 ± 0.07 | 0.81 ± 0.04 | 1.53 ± 0.10 | 0.99 ± 0.06 |
| 35 | 7.20 ± 0.63 | 3.78 ± 0.28 | 4.23 ± 0.32 | 1.00 ± 0.06 | 3.68 ± 0.28 | 5.57 ± 0.41 | 2.94 ± 0.18 | 1.02 ± 0.07 | 0.75 ± 0.04 | 0.93 ± 0.06 | 1.06 ± 0.06 |
| 36 | 5.37 ± 0.41 | 3.53 ± 0.24 | 2.59 ± 0.14 | 0.88 ± 0.05 | 1.98 ± 0.13 | 5.26 ± 0.39 | 3.25 ± 0.20 | 0.98 ± 0.07 | 0.70 ± 0.04 | 1.46 ± 0.11 | 0.84 ± 0.05 |
| 37 | 7.61 ± 0.58 | 3.61 ± 0.24 | 2.30 ± 0.46 | 0.76 ± 0.05 | 1.68 ± 0.12 | 4.67 ± 0.35 | 2.77 ± 0.16 | 1.23 ± 0.09 | 0.75 ± 0.04 | 1.15 ± 0.09 | 0.93 ± 0.05 |
| 38 | 5.81 ± 0.43 | 2.68 ± 0.23 | 4.57 ± 0.33 | 1.20 ± 0.09 | 4.48 ± 0.32 | 5.57 ± 0.40 | 3.06 ± 0.21 | 0.99 ± 0.06 | 0.86 ± 0.04 | 1.63 ± 0.11 | 0.99 ± 0.06 |
| 39 | 5.14 ± 0.38 | 4.03 ± 0.29 | 3.37 ± 0.21 | 1.01 ± 0.08 | 2.44 ± 0.17 | 5.77 ± 0.42 | 3.53 ± 0.24 | 0.81 ± 0.05 | 0.69 ± 0.03 | 1.07 ± 0.06 | 0.82 ± 0.05 |
| 40 | 3.98 ± 0.28 | 2.44 ± 0.18 | 1.65 ± 0.08 | 0.84 ± 0.05 | 0.95 ± 0.05 | 5.56 ± 0.42 | 4.74 ± 0.33 | 1.34 ± 0.10 | 0.40 ± 0.02 | 1.56 ± 0.11 | 0.69 ± 0.04 |
| 41 | 3.68 ± 0.25 | 2.28 ± 0.15 | 2.53 ± 0.15 | 1.19 ± 0.09 | 0.78 ± 0.04 | 4.96 ± 0.36 | 5.36 ± 0.42 | 1.15 ± 0.08 | 0.32 ± 0.02 | 0.92 ± 0.07 | 0.59 ± 0.03 |
| 42 | 7.89 ± 0.65 | 3.69 ± 0.24 | 2.30 ± 0.12 | 0.72 ± 0.04 | 1.56 ± 0.09 | 5.08 ± 0.36 | 2.58 ± 0.15 | 0.91 ± 0.06 | 0.96 ± 0.05 | 1.03 ± 0.06 | 0.96 ± 0.05 |
| 43 | 5.63 ± 0.44 | 2.92 ± 0.19 | 2.61 ± 0.15 | 0.93 ± 0.06 | 1.82 ± 0.09 | 5.83 ± 0.49 | 3.61 ± 0.21 | 1.18 ± 0.08 | 0.67 ± 0.04 | 0.92 ± 0.06 | 0.93 ± 0.06 |
| 44 | 5.08 ± 0.40 | 2.68 ± 0.18 | 7.58 ± 0.65 | 1.50 ± 0.10 | 8.15 ± 0.69 | 4.80 ± 0.34 | 4.18 ± 0.30 | 0.99 ± 0.07 | 1.22 ± 0.09 | 1.38 ± 0.08 | 1.07 ± 0.08 |
| 45 | 7.18 ± 0.62 | 3.65 ± 0.22 | 3.19 ± 0.25 | 0.96 ± 0.06 | 1.96 ± 0.10 | 5.14 ± 0.35 | 3.50 ± 0.20 | 0.83 ± 0.05 | 0.84 ± 0.05 | 1.36 ± 0.09 | 0.92 ± 0.06 |
| 46 | 6.82 ± 0.55 | 3.33 ± 0.20 | 3.32 ± 0.25 | 1.01 ± 0.07 | 2.58 ± 0.14 | 4.70 ± 0.33 | 3.42 ± 0.21 | 1.10 ± 0.08 | 0.86 ± 0.05 | 1.34 ± 0.09 | 0.93 ± 0.06 |
| 47 | 3.79 ± 0.24 | 3.02 ± 0.18 | 2.88 ± 0.17 | 1.10 ± 0.09 | 2.12 ± 0.14 | 5.35 ± 0.40 | 4.76 ± 0.35 | 1.15 ± 0.09 | 0.34 ± 0.02 | 1.46 ± 0.10 | 0.78 ± 0.04 |
| 48 | 7.34 ± 0.61 | 2.28 ± 0.11 | 2.86 ± 0.16 | 0.93 ± 0.07 | 3.02 ± 0.22 | 4.71 ± 0.38 | 3.31 ± 0.21 | 1.28 ± 0.10 | 0.63 ± 0.04 | 1.42 ± 0.09 | 0.95 ± 0.04 |
| 49 | 5.92 ± 0.48 | 2.75 ± 0.16 | 2.80 ± 0.17 | 0.99 ± 0.08 | 1.96 ± 0.15 | 4.54 ± 0.36 | 3.67 ± 0.23 | 0.81 ± 0.05 | 0.68 ± 0.03 | 1.02 ± 0.06 | 0.82 ± 0.05 |
| 50 | 5.01 ± 0.45 | 2.34 ± 0.17 | 1.83 ± 0.10 | 0.83 ± 0.06 | 1.41 ± 0.10 | 5.51 ± 0.45 | 3.21 ± 0.21 | 1.10 ± 0.07 | 0.61 ± 0.04 | 1.32 ± 0.08 | 0.80 ± 0.05 |

Table 3 Composition of the modified Hoagland solution

| Name | Concentration |
| --- | --- |
| KNO_3_ | 10 (High N) or 1 (Low N) mM |
| KH_2_PO_4_ | 0.04 mM |
| K_2_HPO_4_ | 0.015 mM |
| KCl | 0.63 mM |
| K_2_SO_4_ | 1 mM |
| MgSO_4_ | 0.5 mM |
| CaCl_2_ | 3 mM |
| Fe-Na EDTA | 0.2 mM |
| H_3_BO_3_ | 14 µM |
| ZnSO_4_ | 3 µM |
| MnSO_4_ | 5 µM |
| CuSO_4_ | 0.7 µM |
| (NH_4_)_6_Mo_7_O_24_ | 0.7 µM |
| CoCl_2_ | 0.1 µM |

Table 4 Primers used in this study

| Genes | Primers for real-time RT-PCR | Reaction program | Amplicon length | Primer amplification efficiencies | Statistical method used for relative expression calculation |
| --- | --- | --- | --- | --- | --- |
| *BnACTIN* | Forward: 5'-ACGAGCTACCTGACGGACAAG-3'  Reverse: 5'-GAGCGACGGCTGGAAGAGTA-3' | 35 cycles  Denaturation at 94°C  Annealing at  65°C  Extension at  72°C | 150 bp | E=10^(-1/slope)×100%-1 | 2^-ΔΔCt^ |
| *BnNRT1.1* | Forward: 5'-ATGGTAACCGAAGTGCCTTG-3'  Reverse: 5'-TGATTCCAGCTGTTGAAGC-3' |  |  |  |  |
| *BnNRT1.5* | Forward: 5'-CAATCTACTTGATCGCATTG-3'  Reverse: 5'-CCTGTAGGCTTGAAGTTTCG-3' |  |  |  |  |
| *BnNRT1.8* | Forward: 5'-GGGTATGGTGGTTATCAGCCC-3'  Reverse: 5'-CGAAAGGAGCGATCCGAGG-3' |  |  |  |  |
| *BnNRT2.1* | Forward: 5'-TGGTGGAATAGGCGGCTCGAGTTG-3'  Reverse: 5'-GTATACGTTTTGGGTCATTGCCAT-3' |  |  |  |  |
| *BnNRT2.5* | Forward: 5'-TTATCGCACGAGAACAAAG-3'  Reverse: 5'-TTCCACAATGGGGAGGTATG-3' |  |  |  |  |
| *BnNRT2.6* | Forward: 5'-TCACGGCAAGGGAACAAAT-3'  Reverse: 5'-AAGGTGGAGATGAAGCAGGT-3' |  |  |  |  |

Fig.1 Regression analysis between root biomass and NUpE (A-B) across the 50 rapeseed genotypes under high and low N levels.

Fig.2 Differences in root diameter (A-B) between N-efficient-genotype and N-inefficient-genotype under hydroponic culture. Each bar above the mean value represents standard error of the mean (n = 3), and different letters above the bars within a figure indicate significant differences according to the ANOVA-protected LSD_0.05_ test.

Fig.3 Differences in superoxide dismutase (SOD; A-B) and soluble sugar (C-D) between N-efficient-genotype and N-inefficient-genotype under hydroponic culture. Each bar above the mean value represents standard error of the mean (n = 3), and different letters above the bars within a figure indicate significant differences according to the ANOVA-protected LSD_0.05_ test.

Fig.4 Differences in root *BnNRT 1.8* (A-B) and *BnNRT 2.6* expression (C-D) between N-efficient-genotype and N-inefficient-genotype under hydroponic culture. Each bar above the mean value represents standard error of the mean (n = 3), and different letters above the bars within a figure indicate significant differences according to the ANOVA-protected LSD_0.05_ test.
